# ﻿Haplotype-resolved genomes of *Phlebopus portentosus* reveal nuclear differentiation, TE-mediated variation, and saprotrophic potential

**DOI:** 10.3897/imafungus.16.161411

**Published:** 2025-08-28

**Authors:** Guoliang Meng, Jiajia Li, Yao Cao, Fan Li, MengQian Liu, Rongchun Li, Caihong Dong

**Affiliations:** 1 State Key Laboratory of Microbial Diversity and Innovative Utilization, Institute of Microbiology, Chinese Academy of Sciences, Beijing, 100101, China Institute of Microbiology, Chinese Academy of Sciences Beijing China; 2 Yunnan Junshijie Biotechnology Ltd., Kunming 650200, Yunnan, China Yunnan Junshijie Biotechnology Ltd. Kunming China

**Keywords:** Genome architecture, *Phlebopus portentosus*, secondary metabolite biosynthesis gene clusters, transposable elements, trophic strategy

## Abstract

*Phlebopus portentosus* is a widely consumed edible mushroom and the only *Boletales* species currently cultivated on an industrial scale. Despite its economic importance, its trophic strategy and genomic adaptations remain elusive. Here, we presented high-quality, chromosome-level genome assemblies for two sexually compatible monokaryons (PP78 and PP85) of *P. portentosus*. Comparative genomic analysis revealed a genome size difference of 1.17 Mb (30.87 vs. 32.04 Mb), primarily attributed to transposable element (TE) expansion in strain PP85. Genome structural variations were largely driven by TEs, particularly LTR retrotransposons. DNA transposons were also involved in structural rearrangement of secondary metabolite biosynthetic gene clusters, impacting their organization and transcriptional profiles. Functional annotation identified 187 PP78-specific and 236 PP85-specific genes, with the latter enriched in TE-related and putative virulence factors. *P. portentosus* displays genomic signatures of both ECM symbiosis (reduced lignocellulose-degrading enzymes) and saprotroph (expanded glycoside hydrolase 31 and sugar transporters), supporting a facultative ECM lifestyle. The expansion of non-ribosomal peptide synthetase and polyketide synthase pathways, alongside contraction of terpenoid clusters typical of ECM fungi, further indicated its adaptation to saprotroph. These findings highlight the role of TEs in driving genome plasticity, metabolic diversity, and nuclear divergence in *P. portentosus*, providing valuable genomic resources for this species.

## ﻿Introduction

*Phlebopus portentosus* (Berk. and Broome) Boedijn, a widely appreciated edible and medicinal mushroom, is the only *Boletales* species currently cultivated on an industrial scale. It is characterized by high protein and low-fat content and is rich in essential amino acids and minerals (Sanmee et al. 2003; Zou et al. 2021). It contains a variety of bioactive compounds, such as polysaccharides, flavonoids, alkaloids, and terpenoids, which are associated with antioxidant (Kaewnarin et al. 2016; Kumla et al. 2021; Li et al. 2024), antitumor (Li et al. 2024), and antimicrobial activities (Kumla et al. 2021; Li et al. 2024). Due to its nutritional profile, pleasant flavor, strong market demand, and high consumption potential, *P. portentosus* is considered a valuable species for food and pharmaceutical applications (Kumla et al. 2011).

Most species of *Boletales* are ectomycorrhizal (ECM) fungi (Wu et al. 2014; Wu et al. 2022). However, the nutritional strategy of *P. portentosus* remains debated. Based on mycorrhizal synthesis and stable isotope analyses, Kumla et al. (2016) proposed an ECM lifestyle for this species, supporting a symbiotrophic mode of nutrition. Other studies reported its inability to form typical ECM structures, such as mantle sheaths and Hartig nets, under in vitro conditions, suggesting a possible loss of its ECM niche in natural environments (Zhang et al. 2015, 2016). The free-living mycelia of *P. portentosus* can produce basidiocarps without the presence of a host tree, indicating its saprotrophic ability. Although *P. portentosus* exhibits certain saprotrophic traits, its nutrient acquisition strategy differs notably from that of classical saprotrophs, raising the possibility that it may be facultatively ECM (Sanmee et al. 2010; Ji et al. 2011; Cao et al. 2021). Given these uncertainties, recent advances in genome sequencing have enabled a more detailed exploration of fungal trophic modes at the genomic level (Van et al. 2015; Miyauchi et al. 2020).

In basidiomycetes, including economically important edible and medicinal species such as *Lentinula edodes*, *Ganoderma lucidum*, *Pleurotus ostreatus*, and *Auricularia heimuer*, the dikaryotic stage dominates the life cycle (Bao 2024﻿). A recent study reported that the haploid set of chromosomes is not uniformly distributed into each nucleus in *Sclerotinia sclerotiorum* and *Botrytis cinerea* (Xu et al. 2025), suggesting genetic differences between nuclei. In sexually compatible strains, each nucleus retains its regulatory machinery while engaging in inter-nuclear communication (Liu et al. 2017; Gehrmann et al. 2018). As a result, haploid genomes alone do not fully capture the species’ genetic complexity. Comparative analyses between the two nuclei in sexually compatible strains enabled detailed investigations of nuclear interactions and gene expression regulation (Gehrmann et al. 2018; Shen et al. 2024). Despite the availability of five published draft genomes of *P. portentosus*, analyses focusing on nuclear-level differences remain lacking, as these genomes did not involve comparisons between the two nuclei in heterokaryotic strains (Cao et al. 2015; Li 2020; Wan et al. 2021; Yang et al. 2024; Kang et al. 2025).

Previous genomic studies confirmed the presence of a relatively high abundance of transposable elements (TEs) in *P. portentosus* (Cao et al. 2015; Li 2020; Wan et al. 2021; Kang et al. 2025). TEs, also known as “jumping genes,” are mobile DNA segments capable of relocating within the genome, often leading to genomic rearrangements, mutations, and increased diversity (Feschotte and Pritham 2007). In eukaryotes, TEs served as major drivers of genome evolution and genetic diversity by mediating structural variations, generating genetic polymorphisms, and reshaping regulatory elements (Nishihara 2019; Pimpinelli and Piacentini 2020; Romano and Fanti 2022). The role of abundant TEs in the genome evolution and nuclear differentiation of *P. portentosus* is the focus of our investigation.

The successful artificial cultivation of *P. portentosus* has sparked a new wave of research interest in this species. In this study, we obtained two sexually compatible monokaryotic strains, PP78 and PP85, through an improved protoplast regeneration method, coupled with microscopic observation and molecular validation. Using PacBio HiFi sequencing and a manually curated assembly pipeline, we generated telomere-to-telomere, chromosome-level genome assemblies for both strains P, representing the highest quality genomes reported for this species to date. Comparative analyses between the two nuclear genomes revealed the roles of TEs in shaping genome evolution, nuclear differentiation and the diversification of secondary metabolite gene clusters. Furthermore, integrative genomic and transcriptomic analyses uncovered features associated with the species’ weak saprotrophic capacity. Our findings shed light on the contributions of TEs to genome plasticity, nuclear divergence and metabolic diversity in *P. portentosus*, while also providing valuable genomic resources for further studies on cultivation optimization and metabolic engineering.

## ﻿Materials and methods

### ﻿Strain isolation, mycelia culture and DNA extraction

The *P. portentosus* strains, Ph5-2 (CGMCC5.2272), Ph7BN1-2, Ph46SM1-3 and Ph64WS2-2 were individually isolated from wild specimens collected in Xishuangbanna, Pu’er, and Wenshan, Yunnan Province, China, by Yao Cao from Yunnan Junshijie Biotechnology Co., Ltd.

Monokaryotic strains were generated using an improved protoplast monokaryotization protocol (Chang et al. 1985) in combination with a solid-liquid biphasic protoplast regeneration method (Reed et al. 2021) from parental strain Ph5-2. Mating types were determined by PCR amplification using self-designed mating-type-specific primers (HD2J-F: GGGAGAACTTYTCWGTCA, HD2J-R: ATACACGTATGGGTAAGC) and paired culture assays. Two sexually compatible strains, PP78 and PP85, were selected for genome sequencing.

For DNA extraction, the dikaryotic and monokaryotic strains were cultured separately in M1 liquid medium (per liter 200 g of potato, 20 g of glucose, 2 g of yeast extract, 1 g of MgSO_4_, and 1 g of KH_2_PO_4_) at 28 °C with shaking at 120 r/min for 10 d, followed by harvesting *via* filtration. The collected mycelia were flash-frozen in liquid nitrogen, ground into fine powder, and subjected to genomic DNA extraction following a standardized protocol.

### ﻿Genome sequencing and heterozygosity estimation

The DNA quality was assessed by 1% agarose gel electrophoresis and quantified using a Qubit2.0® Fluorometer (Life Technologies, New York, USA). DNA samples from strains PP78 and PP85 were sequenced on the MGISEQ-T7 (with a 350 bp library) and the PacBio Sequel II (with a 15 Kb SMRTbell library) platforms at Wuhan Grandomics Biosciences Co., Ltd. (Wuhan, Hubei, China).

To remove low-quality reads, adapter sequences, and reads containing poly-N, the raw paired-end reads from MGISEQ-T7 sequencing were preprocessed using fastp v.0.20.0 (Chen et al. 2018) with default parameters. Genome heterozygosity was then estimated through K-mer distribution analysis using Jellyfish v.2.1.3 (Marcais and Kingsford 2011) based on the MGISEQ-T7 reads. The results were subsequently imported into GenomeScope v.1.0 (Vurture et al. 2017), using a K-mer length of 21 and a ploidy setting of 2, as well as GenomeScope v.2.0 (Ranallo-Benavidez et al. 2020) with the same k-mer length but a ploidy setting of 1.

### ﻿Genome assembly and assessment

The genome contigs were *de novo* assembled using HiFi reads with two software: Hifiasm v.0.16.1-r375 (Cheng et al. 2021) and HiCanu v.2.29 (Nurk et al. 2020), both with default parameters. The initial assemblies were filtered to remove organellar contigs by comparing against the mitochondrial genome. The two assembled genome versions were then aligned using MUMmer v.4.0.0.rc1 (Marçais et al. 2018) to merge partially incomplete contigs. For contigs lacking complete telomeric sequences, terminal sequences and unaligned contigs, telomeric repeat sequences ‘CCCTAA’ and ‘TTAGGG’ were extracted. These sequences were then extended using HiFi assembly with Hifiasm v.0.16.1-r375 (Cheng et al. 2021) by iterative alignment to existing contigs until overlaps were achieved. Further polishing of the assembly was conducted using Racon (Vaser et al. 2017) and Pilon (Walker et al. 2014).

The raw sequencing data were then aligned to the assembled genome using minimap2 v.2.1 (Li 2018). Average coverage depth was calculated with Sambamba v.0.6.6 (Tarasov et al. 2015) using a sliding window of 10 Kb. Data visualization was performed with the Integrative Genomics Viewer (IGV) (Thorvaldsdóttir et al. 2013), enabling the identification of local coverage anomalies. Regions displaying coverage anomalies were manually adjusted by re-examining the PacBio HiFi reads. This iterative process resulted in a high-quality, chromosome-level genome assembly.

To evaluate the completeness of the genome assembly, Compleasm v.0.2.6 (Huang and Li 2023) was employed using the basidiomycota_odb10 lineage dataset. Additionally, MGI and HiFi reads were aligned to the genome using BWA v.0.7.17-r1188 (Li and Durbin 2009) and minimap2 v.2.1 (Li 2018) to assess assembly integrity based on mapping rates. The accuracy of the final assembly was further estimated through k-mers mapping with Merqury v.1.3 (Rhie et al. 2020). Genome continuity was assessed based on the N50 values, LTR assembly index (LAI) scores (Ou and Jiang 2018), and coverage depths.

### ﻿Genome annotation

The final assembly’s *de novo* repeat library was constructed using RepeatModeler v.2.0.1 (Flynn et al. 2020). Transposable elements (TEs) were identified and masked with RepeatModeler v.2.0.1 (Flynn et al. 2020) and RepeatMasker v.4.0.9 (Tarailo-Graovac and Chen 2009). Further analysis of LTR retrotransposons was performed using LTR_finder (Xu and Wang 2007), LTRharvest (Ellinghaus et al. 2008) and LTR_retriever (Ou and Jiang 2018) for *de novo* identification. Unknown TEs were classified based on the fungal model in DeepTE (Yan et al. 2020) with default parameters. Tandem repeats were identified using Tandem Repeats Finder (TRF) v.4.09.1 (Benson 1999) with default settings.

Gene prediction and functional annotation were carried out using the Funannotate v.1.7.4 pipeline (Palmer and Stajich 2018). After cleaning, sorting/renaming headers, and repeat-masking the assembly, genome annotation was performed by integrating evidence from *ab initio* gene prediction, homology-based annotation, and transcriptome data (Suppl. material 1: tables S1, S2). *Ab initio* gene prediction was conducted using AUGUSTUS v.3.3.3 (Stanke and Waack 2003), SNAP (Korf 2004), GlimmerHMM (Salzberg et al. 1999) and GeneMark-ES (Lomsadze 2005), with *Coprinus cinereus* selected as the reference species. For homology-based gene prediction, candidate reference species were assessed for genome and proteome completeness, and those with ≥ 95% completeness were selected. Four *Boletales* species (Suppl. material 1: table S2) were chosen as reference protein sets and analyzed using GeMoMa v.1.6.2 (Keilwagen et al. 2016) with default parameters. Consensus gene models were generated by Funannotate v.1.7.4 pipeline (Palmer and Stajich 2018) with EVidenceModeler v.1.1.1 (Haas et al. 2008). Protein integrity was evaluated using Compleasm v.0.2.6 (Huang and Li 2023) based on the basidiomycota_odb10 lineage dataset.

Predicted protein sequences were aligned against the Swiss-Prot, Pfam, BUSCO, MEROPS, and dbCAN databases using DIAMOND v.0.9.24.125 (Buchfink et al. 2015), with an E-value threshold of ≤ 1e-5. Additional annotations including Gene Ontology (GO), Clusters of Orthologous Groups (COG), and Kyoto Encyclopedia of Genes and Genomes (KEGG), were performed using InterProScan v.5.0 (Philip et al. 2014) and eggNOG-mapper v.2 (Cantalapiedra et al. 2021) with default parameters.

Secondary metabolite gene clusters were predicted with fungal AntiSMASH v.3.0 (https://fungismash.secondarymetabolites.org/, accessed on February 11, 2023). Identification of strain-specific genes in two sexually compatible strains was performed using OrthoFinder v.2.5.4 (Emms and Kelly 2015).

### ﻿Phylogenomic and evolutionary analyses

High-quality genomes of 16 *Boletales* species (Suppl. material 1: table S4), along with 3 *Atheliales* species and two fossil-record fungal species (*C. cinerea* and *Laccaria bicolor*), were selected for phylogenomic analysis (Floudas et al. 2012). Single-copy orthologous genes were identified using OrthoFinder v.2.5.4 (Emms and Kelly 2015). Sequence alignment and trimming were conducted with MAFFT v.7.505 (Katoh et al. 2002) and Gblocks v.0.91b (Castresana 2000) *via* PhyloSuite v.1.2.3 (Zhang et al. 2020), using default parameters. The processed sequences were concatenated, and the best-fit model was determined using ModelFinder (Kalyaanamoorthy et al. 2017).

A maximum likelihood phylogenetic tree was subsequently constructed using RAxML-NG v.0.9.0 (Kozlov et al. 2019), with the JTT+F+I+G4 model identified as the best fit. The resulting phylogenetic tree was visualized in FigTree v.1.4.4 (https://github.com/rambaut/figtree/). Species divergence times were estimated using BEAST v.2.67 (Bouckaert et al. 2014), calibrated with the fossil records from *C. cinerea* and *L. bicolor*. Phylogenetic relationships and gene family data were further analyzed to assess gene family expansions and contractions using CAFE v.5.2.1 (De et al. 2006). Gene families exhibiting significant expansion or contraction were identified with a threshold of *P* < 0.05. Finally, KEGG and GO functional enrichment analyses were performed to explore the biological relevance of these gene families.

### ﻿Putative Carbohydrate-active enzyme (CAZyme)-encoding genes

Based on the established nutritional types of *Basidiomycota* species (Floudas et al. 2012; Wu et al. 2022), protein prediction data for species with available results were retrieved from the NCBI and JGI databases (Suppl. material 1: table S3). Protein integrity was assessed using Compleasm v.0.2.6 (Huang and Li 2023) based on the basidiomycota_odb10 lineage dataset, and only species with > 95% completeness were retained for further analysis of major nutritional modes. Functional annotation of carbohydrate-active enzymes (CAZymes) was conducted using the dbCAN2 meta server (Zhang et al. 2018), combining results from three methods: HMMER+dbCAN (E-value threshold of < 1e-15, coverage > 0.35), DIAMOND+CAZy (E-value threshold of < 1e-102), and Hotpep+PPR (conserved peptide hits > 6, cumulative peptide frequency > 2.6). Only candidates supported by at least two methods were considered reliable. A heatmap was generated to visualize the abundance patterns of various CAZyme families and to perform hierarchical clustering based on their distribution profiles.

### ﻿Comparative genomic analysis

Whole-genome synteny analysis was performed on the monoploid genomes of strains PP78 and PP85 using MUMmer v.4.0.0.rc1 (Marçais et al. 2018). To examine gene collinearity relationships, MCScanX (Wang et al. 2012) was used with an E-value threshold of ≤ 1e-5, enabling the identification of syntenic and collinear genes. Synteny across multiple genomes was visualized using NGenomeSyn (He et al. 2023). In addition, SyRI v.1.5 software (Goel et al. 2019) was used to detect structural variations greater than 10 Kb between homologous chromosomes. Unique genes between the genomes of strains PP78 and PP85 were identified using blastp with an E-value threshold of ≤ 1e-5.

### ﻿Transcriptome analysis

In this study, nine RNA-seq libraries representing the developmental stages of mycelium, primordium, and fruiting body of *P. portentosus* were retrieved from publicly available databases (Suppl. material 1: table S1). The processed RNA-seq reads were initially aligned to the assembled genomes of strains PP78 and PP85 using HISAT2 v.2.2.1 (Kim et al. 2019). Transcript abundance, expressed as transcripts per million (TPM), was subsequently quantified using featureCounts v.2.0.6 (Liao et al. 2014).

### ﻿Quantitative real-time PCR analysis

Total RNA extraction and quality assessment were performed following the method described by Li et al. (2019). cDNA was synthesized from 1 μg of total RNA in a final volume of 20 μL using the All-in-One First-Strand Synthesis MasterMix Kit (LABLEAD Trading Co. Ltd., Beijing, China) according to the provided protocol. The cDNA obtained was diluted 10-fold using nuclease-free water for further Quantitative real-time PCR (qRT-PCR). qRT-PCR was performed to validate the transcriptome data and assess gene expression levels with Taq SYBR® Green qPCR Premix (LABLEAD Trading Co. Ltd., Beijing, China), following the method described by Hu et al. (2023). Reactions were performed on a Bio-Rad CFX Connect Real-Time PCR Detection System (Bio-Rad Laboratories, Inc. California, USA). The SYB gene (*PP78_010542*, *PP85_010848*) was used as the internal reference (Hu et al. 2023). Relative gene expression levels were calculated using the 2^−∆∆Ct^ method. Each experiment included three biological replicates, with two technical replicates. All primer sequences used in this study were listed in Suppl. material 1: table S16.

### ﻿Data analyses

All statistical analyses were performed using GraphPad Prism 8.0 (GraphPad Software Inc., CA, USA) and IBM SPSS 16.0 (SPSS Inc., IL, USA). Statistical significance between two groups was assessed using Student’s *t*-test, while one-way ANOVA followed by multiple range tests was employed for comparisons involving more than two groups.

### ﻿Abbreviations

**TE** Transposable element

**ECM** Ectomycorrhizal

**IGV** Integrative Genomics Viewer

**LAI** LTR assembly index

**CAZyme** Carbohydrate-active enzyme

**TPM** Transcripts per million

**NGS** Next-generation sequencing

**Mya** Million years ago

**HET** Heterokaryon incompatibility

**smBGCs** Secondary metabolite biosynthetic gene clusters

**NRPS** Non-ribosomal peptide synthetases

**T1PKS** Type I polyketide synthases

**PCWDs** Plant cell wall degrading enzymes

## ﻿Results

### ﻿Acquisition of two sexually compatible strains of *Phlebopus portentosus*

**Figure 1.**
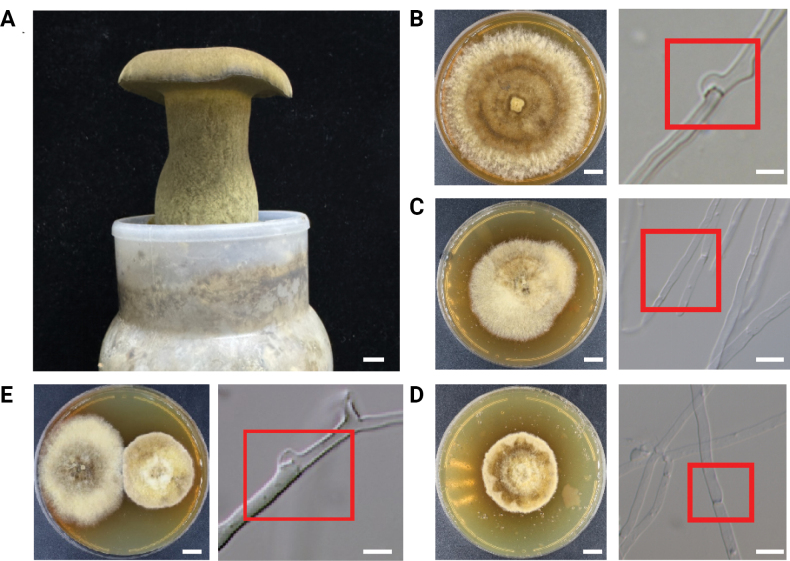
The fruiting body of *Phlebopus portentosus* and the monokaryotic strains used for genome sequencing: **A** The fruiting body of *P. portentosus*. **B** Colony and hyphae of parental strain Ph5-2 with clamp connections. **C** Colony and hyphae of monokaryotic strain PP85. **D** Colony and hyphae of monokaryotic strain PP78. **E** Colony and hyphae resulting from hybridization of the compatible monokaryotic strains PP78 and PP85. The red box indicates the formation or absence of clamp connections at the hyphal septa. Scale bars: 1 cm (fruiting bodies); 1 cm (colony); 2 μm (microscopic observation: **B–E**).

Two monokaryotic strains, PP78 and PP85, were isolated from the parental strain Ph5-2 through protoplast monokaryotization (Fig. 1A–E, Suppl. material 2: fig. S3). PCR amplification using mating-type-specific primers confirmed that these two strains possessed different mating types (Suppl. material 2: fig. S1). They could form a clamp connection when co-cultured (Fig. 1E). Next-generation sequencing (NGS) analysis revealed low heterozygosity levels (PP78: 0.0219%; PP85: 0.0139%), further confirming their monokaryotic nature (Suppl. material 2: fig. S2A, B). Moreover, mating-type gene analysis provided additional evidence of their sexual compatibility as monokaryotic strains (Suppl. material 2: fig. S2C, D).

### ﻿Genome assembly of two sexually compatible strains

Genome sequencing yielded 285,691 HiFi reads (5.17 Gb, ~167.74× genome coverage) for PP78 and 204,296 (3.63 Gb, ~113.48× genome coverage) for PP85. The rDNA regions, located near the telomeres in the *P. portentosus* genome were corrected through targeted read extraction and assembly. The final genome assemblies of the sexually compatible strains PP78 and PP85 were 30.87 Mb (30,874,889 bp) and 32.04 Mb (32,036,162 bp) in size, respectively, each comprising 11 chromosomes and a single circular mitochondrial genome (Suppl. material 1: table S5, Suppl. material 2: fig. S4A). Both genomes contained 22 telomeres, located at the termini of all 11 chromosomes and characterized by (CCCTAA)n and (TTAGGG)n repeat sequences. The assemblies achieved N50 values of 2.62 Mb for PP78 and 3.06 Mb for PP85 (Table 1). The GC content of the PP78 genome was 49.04%, while that of PP85 was 48.92%. Chromosome lengths ranged from 1.78 Mb to 5.00 Mb (Suppl. material 1: table S6). The chromosomes were designated Chr01 to Chr11 in descending order of length, independently for each strain.

**Table 1.** Comparison of genomic information between different *Phlebopus portentosus* strains.

Strains	PP78	PP85	PP17026	PP33
Number of contigs	12	12	62	108
Number of chromosomes	11	11	-	-
Assembly length	30.87	32.04	32.74	30.35
Total N counts	0	0	0	97,661
Contig N50 (Mb)	2.62	3.06	1.26	1.45
Max length (Mb)	3.59	5.00	3.68	3.47
Mean length (Mb)	2.57	2.67	0.53	0.045
Repeat content (%)	19.11	22.31	21.48	17.38
GC (%)	49.04	48.92	48.92	48.86
Assembly level	Chromosome-level	Chromosome-level	Contig	Scaffold
Telomeres	22	22	Unknown	Unknown
BUSCOs (%)	99.60	99.60	95.58	99.43
LAI	23.72	20.66	14.07	12.08
Mapping rate NGS (%)	99.99	99.98	-	-
Mapping rate TGS (%)	99.99	99.63	-	-
Base accuracy (%)	99.9998	99.9997	-	-

NGS: Next-generation sequencing; TGS: third-generation sequencing.

Mapping HiFi reads to the reference genomes confirmed the high continuity of the assemblies (Suppl. material 2: fig. S4B). Genome completeness was supported by high BUSCO scores for both PP78 (99.60%) and PP85 (99.60%) (Suppl. material 1: table S7), excellent alignment rates for both NGS (PP78: 99.99%, PP85: 99.98%) and TGS (PP78: 99.99%, PP85: 99.63%), along with robust LAI scores (PP78: 23.72, PP85: 20.66). Assembly accuracy was further confirmed by QV values of 56.62 for PP78 and 55.96 for PP85, with overall base accuracy estimates of 99.9998% and 99.9997%, respectively (Table 1). These results demonstrate the exceptional quality, accuracy, and reliability of the PP78 and PP85 reference genome assemblies.

A comparative analysis was conducted using previously published genomes of *P. portentosus* strains PP17026 (GenBank: GCA_020232755.1, Wan et al. 2021) and PP33 (GCA_000766925.2, Cao et al. 2015), other genomes were excluded from the study due to the lack of publicly accessible data and related limitations. These earlier assemblies exhibited notably shorter N50 values, with over 1 Mb shorter than those reported in this study. Additionally, the PP33 genome contained a substantial proportion of ambiguous bases (Ns) (Table 1). Collinearity analysis revealed strong alignment between the larger contigs of PP17026 and the genomes of PP78 and PP85 (Suppl. material 2: fig. S5). Further comparisons based on LAI scores and BUSCO completeness confirmed that the PP78 and PP85 assemblies were more complete than the published genomes (Table 1). Moreover, transcriptome data from strains PP33 and PP17026 (Suppl. material 1: table S1) were mapped to each genome, consistently revealing higher alignment rates for the PP78 and PP85 genomes across all developmental stages (Suppl. material 1: table S10). Overall, the PP78 and PP85 assemblies represented the most complete and highest-quality *P. portentosus* reference genomes available to date.

### ﻿Gene prediction and functional annotation

Repetitive sequence prediction identified 5.89 Mb and 7.14 Mb of repeat elements, accounting for 19.11% and 22.31% of the PP78 and PP85 genomes, respectively (Table 2). As in most fungal species, long terminal repeat retrotransposons (LTR-RTs) represented the largest proportion of annotated repetitive elements, comprising approximately 8.99% and 11.55% of the PP78 and PP85 genomes, respectively. Among these, *Gypsy* retrotransposons predominated over *Copia* elements (Table 2). The substantial difference in LTR-RTs content between the two strains was primarily attributed to a greater abundance of *Copia* elements in PP85 (1.48 Mb), which exceeded that of PP78 (345 Kb) by 1.14 Mb. Additionally, LINEs such as *CR1* and *RTE-BovB* elements, along with the DNA transposon Academ-2, were exclusive to PP78, while *RTE-X* elements were found only in PP85.

**Table 2.** Classification of the repeat sequences in the genome of PP78 and PP85.

Classification	Order	Super family	PP78			PP85		
			Number of elements	Length (bp)	Percentage (%)	Number of elements	Length (bp)	Percentage (%)
Class I (Retroelements)			4,402	3,264,757	10.59	4,997	4,046,120	12.65
	SINEs		397	84,273	0.27	444	105,259	0.33
	LINEs		313	369,378	1.20	259	222,944	0.70
		* Penelope *	207	212,887	0.69	104	128,178	0.40
		*CR1*	51	47,808	0.16	0	0	0.00
		*RTE-BovB*	22	69,202	0.22	0	0	0.00
		*RTE-X*	0	0	0	117	68,709	0.21
		*L1*	30	31,949	0.10	36	22,210	0.07
		*I*	1	1,930	0.01	2	3,847	0.01
		Unclassified	2	5,602	0.02	0	0	0.00
	LTRs		3,578	2,771,346	8.99	4,201	3,696,211	11.55
		*PLE*	31	33,162	0.11	30	28,843	0.09
		* Copia *	830	345,629	1.12	1,382	1,484,979	4.64
		* Gypsy *	2,548	2,325,292	7.54	2,617	2,113,505	6.61
		* Ngaro *	9	9,611	0.03	34	15,327	0.05
		Unclassified	160	57,652	0.19	138	53,557	0.17
	Unclassified		114	39,760	0.13	93	21,706	0.07
Class II (DNA transposons)			2,786	2,190,324	7.10	3,001	2,581,528	8.07
	Helitron		49	39,559	0.13	98	92,797	0.29
	CACTA		86	112,316	0.36	76	148,870	0.47
	IS3EU		11	7,364	0.02	34	30,385	0.09
	Mutator		231	258,193	0.84	267	335,864	1.05
	Zisupton		66	213,938	0.69	88	238,242	0.74
	CMC-EnSpm		5	5,806	0.02	20	23,354	0.07
	hAT		209	100,844	0.33	317	176,624	0.55
	Harbinger		314	244,874	0.79	292	310,864	0.97
	Academ-2		98	93,040	0.30	0	0	0.00
	TcMar		924	686,708	2.23	964	673,354	2.10
	Unclassified		793	427,682	1.39	845	551,174	1.72
Unclassified			611	328,143	1.06	596	313,930	0.98
Total TEs			7,799	5,749,147	18.65	8,594	6,941,578	21.70
Rolling-circles			42	33,449	0.11	79	83,926	0.26
Simple repeats			2,202	94,197	0.31	2,259	96,391	0.30
Low complexity			276	13,611	0.04	299	14,514	0.05
Total repeats			10,319	5,890,404	19.11	11,231	7,136,409	22.31

After masking repetitive sequences and removing unsuitable gene models (e.g., those encoding proteins shorter than 50 amino acids or classified as TEs), a total of 10,702 and 10,982 protein-coding genes were predicted in the PP78 and PP85 genomes, respectively, using a multi-strategy approach (Suppl. material 1: table S8). Both genomes also contained 96 transfer RNA (tRNA) genes. Functional annotation using the NR, BUSCO, PFAM, InterPro, EggNog, and COG databases, supplemented by manual curation, resulted in 7,873 genes in PP78 and 7,987 genes with reliable functional annotations (Suppl. material 1: table S9, Suppl. material 2: fig. S6).

**Figure 2.**
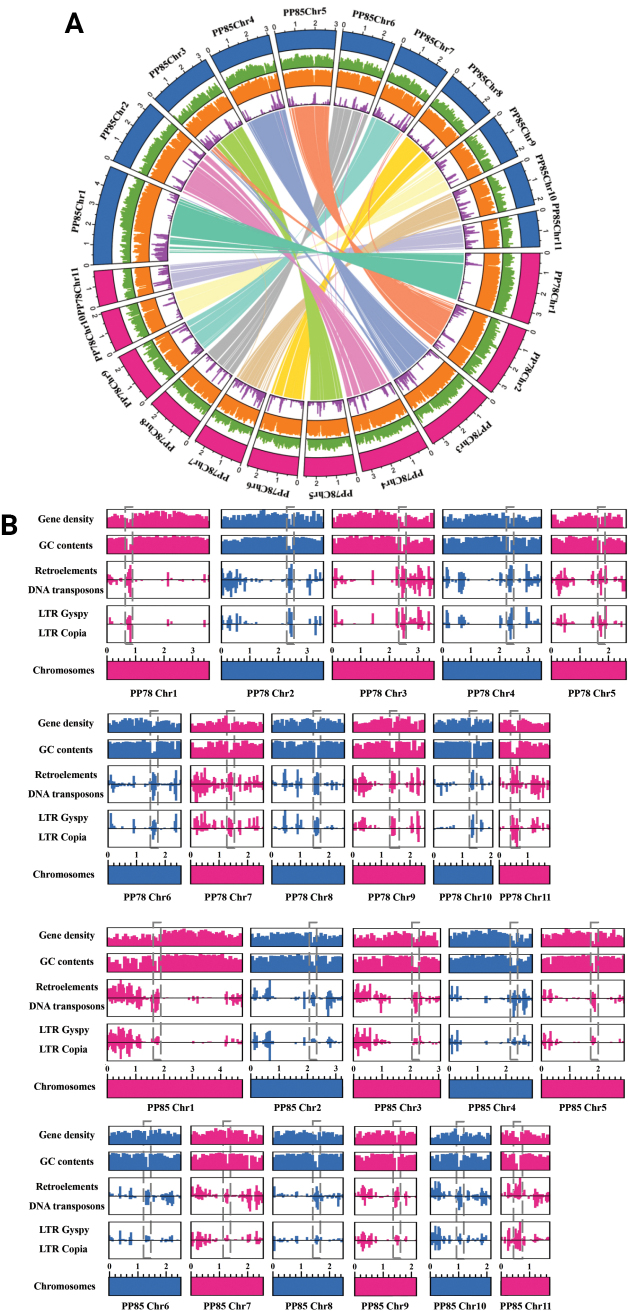
Genomic features and centromere prediction of sexual compatible strains in *Phlebopus portentosus*: **A** Genome circos plot. Blue represents PP85 chromosome; Pink represents PP78 chromosome; Green represents gene density; orange represents GC content; purple represents repeat sequences; The inner circle represents large segmental duplications. **B** Chromosome centromere prediction and characteristic analysis. Gray boxes indicate predicted centromeric regions.

In both assemblies, repetitive sequences were evenly distributed across all chromosomes but were primarily concentrated at the chromosomal ends, while genes were uniformly dispersed throughout the genome (Fig. 2A). By analyzing the distribution patterns of retrotransposons, DNA transposons, and LTR *Gypsy* and *Copia* elements, putative centromeric regions were inferred for each chromosome in both nuclei (Fig. 2B).

### ﻿Phylogenetic analysis

Twenty-one species from the orders *Agaricales* and *Atheliales*, along with two fossil-recorded fungal species (*C. cinerea* and *L. bicolor*), were selected based on stringent criteria for protein completeness (BUSCO > 95%), ensuring high-quality genome data for robust phylogenetic inference (Suppl. material 1: table S4). Among them, a congeneric species *Phlebopus* sp., was found to be genetically distinct from *P. portentosus*, with ITS sequence analysis suggesting it may represent a novel species. Phylogenetic analysis confirmed the placement of *P. portentosus* within the family *Boletinellaceae*, clustering closely with *Phlebopus* sp., consistent with its current taxonomic assignment and evolutionary affinities within *Boletales*. Earlier multi-gene phylogenetic studies (ITS, LSU and *tef*1) first established the placement of the *Phlebopus* genus in *Boletinellaceae*, and proposed a sister-group relationship between *Boletinellaceae* and *Boletaceae* (Wu et al. 2014). A subsequent genomic analysis of 28 *Boletales* species reaffirmed the phylogenetic position of *P. portentosus* within *Boletinellaceae* and the *Phlebopus* clade (Wu et al. 2022). Our phylogenomic analysis supported the current classification of and its evolutionary relationships with other members of *Boletales*. Divergence time analysis indicated that PP78 and PP85 diverged from their closest relatives *Phlebopus* sp. approximately 14–29 million years ago (Mya).

**Figure 3.**
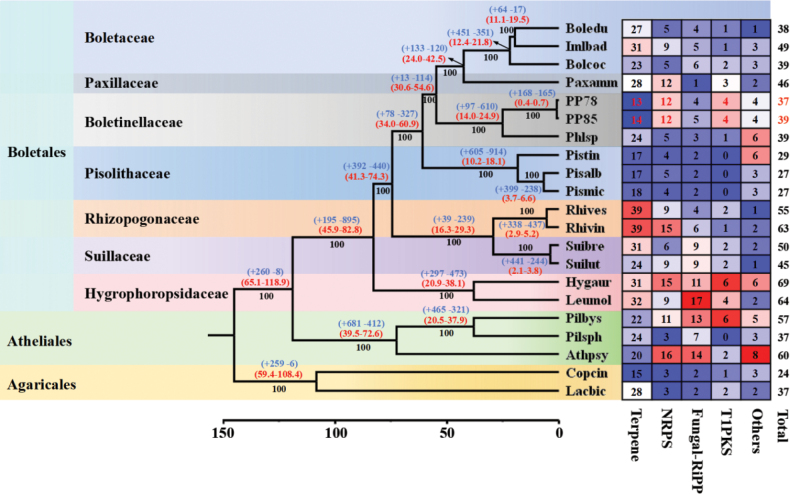
Phylogenetic analysis and gene family evolution of *Phlebopus portentosus* Bootstrap values are indicated in black, divergence time (Mya) with 95% CI (confidence interval) in red, and gene family expansion/contraction in blue.

Gene family analysis revealed 168 expanded and 165 contracted families in *P. portentosus* compared to its close relative *Phlebopus* sp. (Fig. 3), which was reported as the dual symbiotrophic/saprotrophic species (Wu et al. 2022). Among these, 43 gene families exhibited significant expansion (*P* < 0.05), while 15 showed significant contraction (Suppl. material 1: table S11). The expanded gene families were primarily associated with metabolism, stress defense, genome plasticity, and signal transduction. The observed expansion of heterokaryon incompatibility (HET) proteins and hAT family dimerization domains may reflect elevated transposon activity, as both gene families are frequently found within or near TE-rich regions (Bastiaans et al. 2014; Paoletti et al. 2016; Dewing et al. 2022). The significantly contracted gene families included kinase-like domain-containing proteins, PIF1-like helicases and DDE-domain-containing proteins, polyubiquitin genes, etc.

### ﻿Comparative genomic analysis of two sexually compatible strains of *Phlebopus portentosus*

Comparative collinearity analysis of the *A* mating-type loci between PP78 and PP85 revealed that the *HD*1 and *HD*2 genes are arranged in a canonical “head-to-head” orientation (Suppl. material 2: fig. S7). The HD1 proteins shared 49.69% amino acid sequence similarity, while HD2 proteins showed 45.63%. At the *B* mating-type locus, nine *PR* genes were identified across both strains. While several PRs (e.g., PR1-PR4 and PR7-PR9) exhibited high amino acid sequence similarity (> 94%), PR5 and PR6 showed only 25.43% identity and were thus considered distinct. Additionally, two *PP* genes were located near the *PR* clusters; one pair was identical between the strains, whereas the other showed no sequence similarity. The observed divergence at both *A* and *B* mating-type loci between PP78 and PP85 (Suppl. material 2: fig. S7) confirms that PP78 and PP85 represent two sexually compatible strains of *P. portentosus*.

The assembled genomes of these two strains exhibited a notable size difference of 1.17 Mb. Comparative analyses revealed substantial disparities in gene count, chromosome length, repetitive sequence content, and transposon composition. PP85 contained 7.14 Mb of repetitive elements, compared to 5.89 Mb in PP78, accounting for much of the size variation.

**Figure 4.**
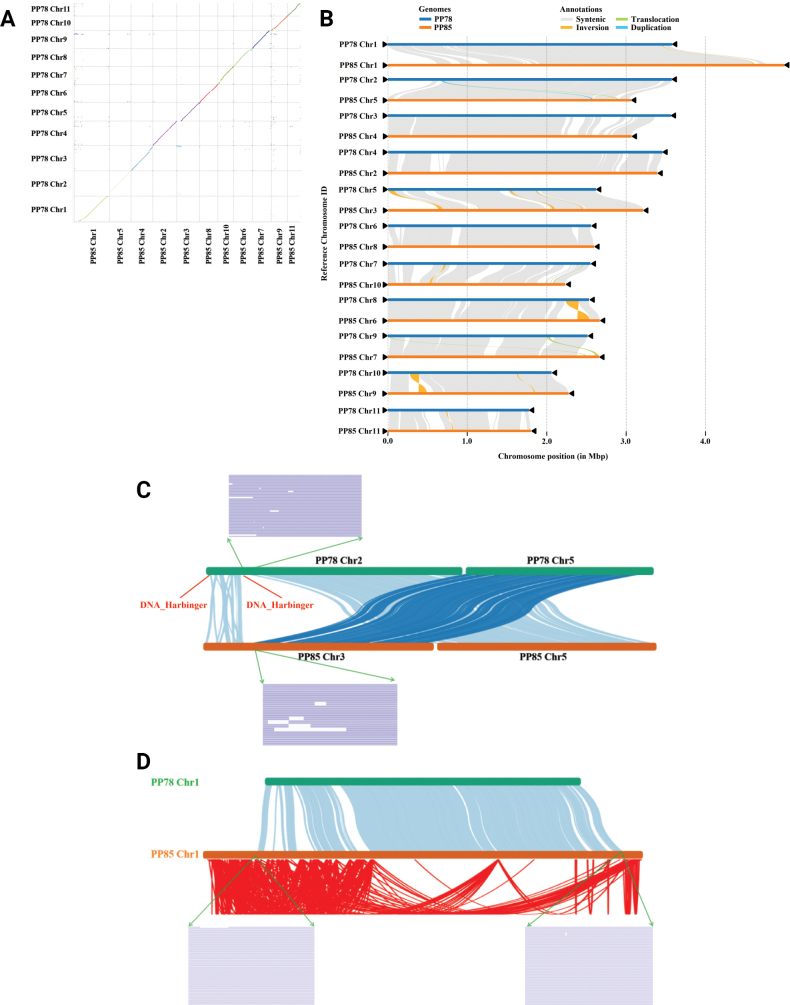
Synteny analysis of the genomes of sexually compatible *Phlebopus portentosus* strains: **A** Dot plot of homologous blocks, where different-colored dots indicate regions homologous to other chromosomal locations in the counterpart genome. The dots with different color represent syntenic regions in homologous chromosome. **B** Chromosome-level local sequence differences. The triangle represents the telomere. **C** Chromosomal structural variation regions between sexually compatible strains; **D** PP78 and PP85 Chr1 synteny analysis. Red indicates intra-chromosomal synteny analysis of PP85 Chr1. Green arrows indicate sequencing reads supporting the structural variation.

Notably, PP85 Chr1 (5.00 Mb) was 1.41 Mb longer than its counterpart in PP78 and had the lowest GC content (47.72%) among all chromosomes in both strains, substantially lower than the 49.71% in PP78 Chr1 (Suppl. material 1: table S6). Similar length discrepancies were observed across other homologous chromosomes. For instance, PP78 Chr3 corresponded to PP85 Chr4, reflecting structural divergence (Fig. 4A). Pearson correlation analysis confirmed a significant association between chromosome length differences and TE content among homologous chromosomes (*P* = 1.2e-6, R = 0.97, Suppl. material 2: fig. S8), implicating TEs as key drivers of genomic divergence.

Synteny analysis using dot plots and collinearity maps demonstrated high conservation across homologous chromosomes (Fig. 4A, B, Suppl. material 2: fig. S9A, B), with collinear regions covering 83.49% of the PP78 genome and 81.90% of PP85 (Suppl. material 1: table S13). Structural variation analysis identified genomic rearrangements, including inversions (493.14 Kb in PP78 vs. 446.46 Kb in PP85), translocations (607.64 Kb in PP78 vs. 582.92 Kb in PP85), and duplications (278.80 Kb in PP78 vs. 586.48 Kb in PP85) (Suppl. material 1: table S12). 175,663 SNPs were identified between the two genomes, while PP85 exhibited more insertions (187.87 Kb) and deletions (161.62 Kb) compared to PP78. Highly diverged regions spanned 3.56 Mb in PP78 and 3.92 Mb in PP85 (Suppl. material 1: table S13).

Large inversions were found within homologous chromosomes, e.g. between PP78 Chr8 and PP85 Chr6, as well as between PP78 Chr10 and PP85 Chr9 (Fig. 4B). Rearrangement also occurred among non-homologous chromosomes. The terminal region of PP78 Chr3 shared high sequence similarity to PP85 Chr3 (Fig. 4A, Suppl. material 2: fig. S9A), and a ~500 Kb fragment from PP78 Chr2 was transferred to non-homologous chromosome PP85 Chr3. Raw sequencing reads confirmed this non-homologous rearrangement, likely mediated by a “cut-and-paste” mechanism involving the DNA_Harbinger transposon (Fig. 4C).

Furthermore, a ~1 Mb region on PP85 Chr1, supported by raw read mapping (Fig. 4D), exhibited no detectable homology to PP78 Chr1 or any other chromosome in either strain (Fig. 4A, B, D). To further confirm the uniqueness of this region, read-depth analysis was performed using high-coverage re-sequencing data (~115×) of the parental dikaryotic strain Ph5-2. PP85-specific segments in Ph5-2 exhibited approximately half the read depth of conserved regions, consistent with their presence in only one of the two nuclei and exclusive inheritance by PP85 (Suppl. material 2: fig. S11A). In contrast, mapping PP78 Chr1 to Ph5-2 yield nearly uniform coverage across the chromosome (Suppl. material 2: fig. S11B).

Using PP85 Chr1 as a reference, mapping re-sequencing data from additional dikaryotic strains (Ph7BN1-2, Ph46SM1-3, and Ph64WS2-2) showed that portions of PP85-specific segments also exhibited approximately half the read depth across different isolates, indicating that this inter-nuclear divergence is not exclusive to Ph5-2 (Suppl. material 2: fig. S12). This unique region of PP85 was 72.34% transposon-rich, with retrotransposons accounting for 64.71%. These findings suggested that retrotransposon proliferation contributed to the expansion of PP85 Chr1 (Fig. 4D).

Moreover, the relatively recent estimated insertion times of transposons (*P* < 0.05, Suppl. material 2: fig. S10) further supported the hypothesis of ongoing transposon-mediated genome remodeling.

By comparing gene sets and defining genes absent in one strain as strain-specific, 187 protein-coding genes unique to PP78 (1.75% of its total protein-coding genes) and 236 unique to PP85 (2.15%) were identified. To validate these findings, we analyzed reads per kilobase of nucleus-specific genes using resequencing data from the parental heterokaryon strain Ph5-2. Strain-specific genes exhibited significantly lower values than shared genes, particularly in PP85, where the median was approximately half that of conserved genes. Using Chr1 of strain PP85 as an example, re-alignment of resequencing data showed reduced reads per kilobase in nucleus-specific genes (Suppl. material 2: fig. S11C). These results support the presence of unique genes and indicate that some genomic differences arise from nuclear separation rather than technical artifacts (Suppl. material 2: fig. S11D). These strain-specific genes were distributed across all chromosomes in both genomes (Suppl. material 2: fig. S13A). Notably, the majority were annotated as hypothetical proteins (91.44% in PP78 and 88.14% in PP85), substantially exceeding the overall proportion of hypothetical proteins in the respective genomes (17.42% in PP78 and 25.95% in PP85).

PP85-specific genes were functionally enriched in TEs (e.g., retrotransposon proteins, DDE endonucleases) and putative virulence factors (Suppl. material 1: table S12). In contrast, PP78 appeared to emphasize genome stability, energy metabolism, and telomere protection, which may help maintain biological functions in stable environments (Suppl. material 1: table S12). This functional complementarity may reflect a co-evolutionary strategy among sexually compatible strains, balancing genome stability with adaptive flexibility.

Transcriptional profiles showed that strain-specific genes in PP78 and PP85 exhibited comparable expression patterns across different developmental stages, with significantly lower expression levels (*P* < 0.05) than those of core genes throughout all stages (Suppl. material 2: fig. S13B–D). Four copies of the orsellinic acid-like gene were identified among the PP85-specific genes, and their presence was confirmed by PCR amplification. However, transcriptional profiling and semi-quantitative analysis revealed that these copies remained transcriptionally silent in both PP85 and Ph5-2 at stages of mycelium (Suppl. material 2: fig. S14).

### ﻿Secondary metabolite biosynthetic gene clusters in two sexually compatible *Phlebopus portentosus* strains

Genome mining of the two sexually compatible *P. portentosus* strains, PP78 and PP85, identified 37 and 39 predicted secondary metabolite biosynthetic gene clusters (smBGCs), respectively, underscoring the considerable biosynthetic capacity of this species. Compared to PP78, PP85 harbored one additional terpene cluster and one extra fungal RiPP-like smBGC (Suppl. material 1: table S14).

A total of 58 and 60 core biosynthetic genes were identified in PP78 and PP85, respectively. Among them, two core genes in PP78 and three in PP85 were transcriptionally silent across all stages. Most core genes exhibited peak expression at the mycelial and fruiting body stages, though several smBGCs displayed stage-specific expression patterns. Specifically, core genes from fungal RiPP-like, non-ribosomal peptide synthetases (NRPS), and hybrid smBGCs were predominantly expressed at the mycelial stage, whereas terpene biosynthesis-related genes were mainly upregulated at the primordium and fruiting body stages (Suppl. material 2: fig. S15).

Both strains contained clusters linked to the known bioactive metabolites, including the sesquiterpenoid (+)-δ-cadinol, the polyketide pigment atromentin, and the triterpenoid clavaric acid. Each strain harbored two smBGCs for (+)-δ-cadinol and two for atromentin, respectively (Suppl. material 2: fig. S16). Clavaric acid, a lanostane-type triterpenoid (Jayasuriya et al. 1998; Huang et al. 2020), functioned as a farnesyltransferase inhibitor disrupting Ras-mediated signaling pathways, and exhibiting potent anticancer activity (Ser et al. 2016). (+)-δ-Cadinol, a cadinane-type sesquiterpenoid, has been reported to possess antimicrobial, antifungal, anti-inflammatory (Chang et al. 2008; Mulyaningsih et al. 2010), and cytotoxic properties, including activity against human breast cancer MCF7 cells (Yap et al. 2017). Atromentin, a polyketide-derived quinone with anticoagulant, antioxidant, and antitumor activities, as also plays a role in ecological adaptations (Khanna et al. 1965; Kim and Lee 2009; Lin and Xu 2020; Seibold et al. 2023). Its derivative, atromentic acid, is the second most abundant pigment in *P. portentosus* (Chuankid et al. 2020).

**Figure 5.**
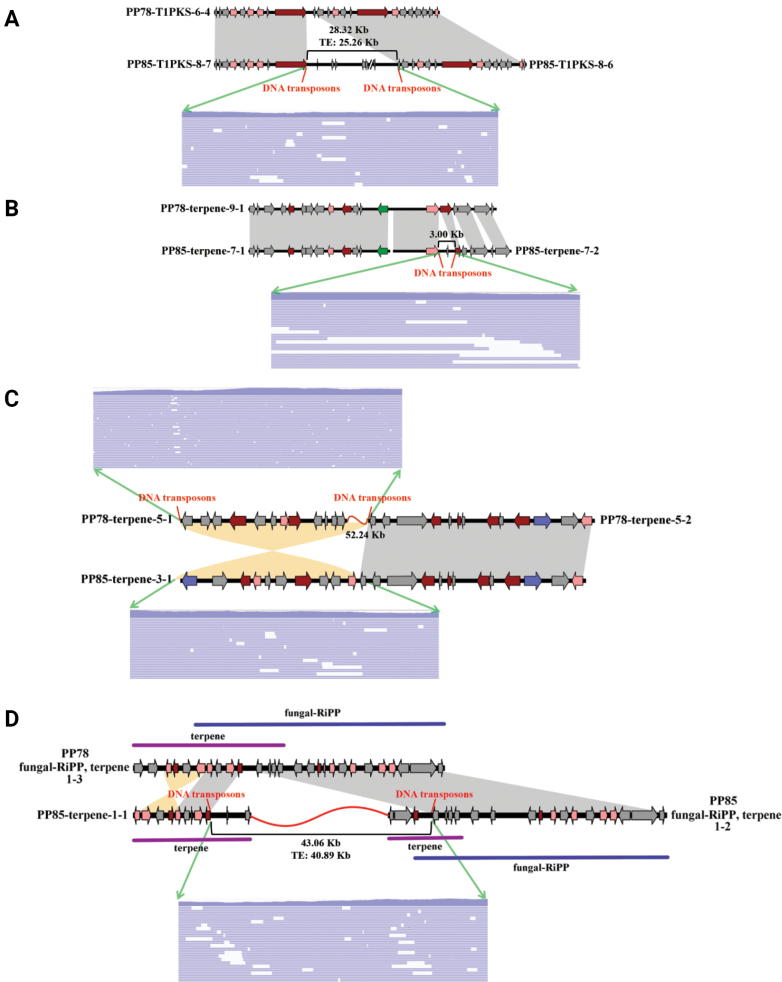
Formation of split secondary metabolite biosynthetic gene cluster in the genomes of sexually compatible *Phlebopus portentosus* strains: **A** Large-scale insertions or deletions *via* “cut-and-paste”, resulting in a T1PKS cluster in strain PP78 corresponding to two distinct clusters in strain PP85. **B** Small fragment insertions or deletions *via* “cut-and-paste”, resulting in a terpene cluster in strain PP78 corresponding to two distinct in strain PP85. **C** Inversions induced by transposon activity, leading to a terpene cluster in strain PP85 corresponding to two distinct clusters in strain PP78. **D** Large-scale insertions or deletions *via* “cut-and-paste”, leading to a fungal-RiPP, terpene cluster in strain PP78 corresponding to a fungal-RiPP and two terpene clusters in strain PP85. Green arrows indicate sequencing reads supporting the structural variation.

Comparative analysis between PP78 and PP85 revealed three distinct structural and organizational patterns of smBGCs. (1) Homologous smBGCs: These were shared between both strains, displaying nearly identical gene content and high sequence similarity, comprising 31 clusters (Suppl. material 2: fig. S17A); (2) Split smBGCs: A single BGC in one strain corresponded to two separate smBGCs in the other, often interrupted by insertions. For instance, terpene clusters 5-1 and 5-2 in PP78 matched a single terpene cluster 3-1 in PP85, separated by a large insertion of 52.24 Kb segment in PP78 (Suppl. material 2: fig. S17B). These structural differences were supported by sequencing reads (Fig. 5); (3) Strain-specific smBGCs: clusters uniquely identified in one strain with only partial gene homology in the other. One such unique cluster was identified in each strain (Suppl. material 2: fig. S17C).

To investigate the mechanisms underlying split smBGCs formation, protein-level synteny analyses were performed, identifying four split smBGCs across two strains, including terpene-, type I polyketide synthases (T1PKS)- and complex fungal-RiPP and terpene-type smBGCs. Integration with transposon annotations suggested that three mechanisms mediated by DNA transposon likely contributed to smBGC reorganization: (1) Large-scale insertions or deletions *via* “cut-and-paste” (Fig. 5A, D). (2) Small fragment insertions or deletions *via* “cut-and-paste” (Fig. 5B). (3) Inversions induced by transposon activity (Fig. 5C).

Expression analysis across different strains revealed significant differences in the transcription levels of homologous genes corresponding to the split gene clusters between PP78 and PP85 (*P* < 0.05, Suppl. material 2: fig. S18). Transposons insertions that caused the division of SmBGCs were associated with elevated transcription levels of genes within smBGCs in PP85 (Suppl. material 2: fig. S18). Although the precise evolutionary direction of these rearrangements remains uncertain, the findings strongly indicate that DNA transposons have shaped the structural diversity of smBGCs in *P. portentosus*.

### ﻿Nutritional strategy of *Phlebopus portentosus*

To investigate the nutritional mode of *P. portentosus*, we conducted a comparative analysis of CAZyme repertoires in strains PP78 and PP85 against those of 46 *Agaricomycetes* species with over 95% genome completeness and well-defined nutritional strategies (Suppl. material 1: table S3, Floudas et al. 2012; Wu et al. 2022). Clustering based on CAZyme profiles grouped *P. portentosus* with symbiotic fungi (Suppl. material 2: fig. S19), consistent with previous studies suggesting a symbiotic tendency (Wan et al. 2021; Wu et al. 2022).

However, a more detailed analysis revealed features indicative of saprotrophic capabilities. *P. portentosus* harbored an unusually high number of GH31 family genes, a large and diverse family that includes glycosidases, lyases, transglycosidases (Arumapperuma et al. 2023) and α-xylosidase (Cao et al. 2020). These enzymes likely play a critical role in the rapid assimilation of carbohydrates during substrate colonization. Among 46 *Agaricomycetes* species, saprotrophs averaged 5.7 GH31 genes, while biotrophs averaged 3.4 (*P* = 0.0005, t-test). Strains PP78 and PP85 possessed 8 and 7 copies, respectively, more closely resembling those of saprotrophic fungi (Suppl. material 1: table S15). Transcriptomic data further showed that these genes were actively transcribed across different developmental stages, suggesting their likely involvement in efficient carbohydrate utilization from cultivation substrates.

**Figure 6.**
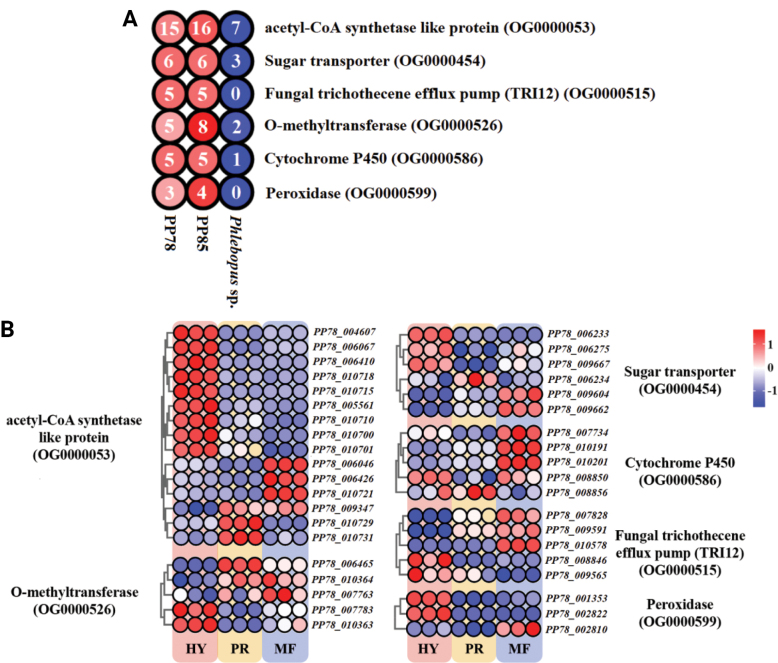
Expanded gene families related with saprotrophy in *Phlebopus portentosus* and their transcriptional profiles: **A** Gene numbers of saprotrophy-related expanded gene families; **B** Transcriptional profiles of saprotrophy-related expanded genes across different developmental stages. HY: hypha; PR: primordium; FB: fruiting bodies; Annotation results reflect the functional trend of the family, not exclusive functions.

Gene family expansion analysis further supported the saprotrophic potential of *P. portentosus*. Compared to its close relative *Phlebopus* sp., gene families associated with acetyl-CoA synthetase-like proteins (OG0000053), sugar transporters (OG0000454), cytochrome P450s (OG0000586), O-methyltransferases (OG0000526), fungal trichothecene efflux pumps (TRI12) (OG0000515), and peroxidases (OG0000599), showed notable expansion (Fig. 6A). All these expanded genes were transcribed across the tested developmental stages. Among these, genes encoding acetyl-CoA synthetase-like proteins, peroxidase, and sugar transporters, were highly expressed during the mycelial stage, likely contributing to the breakdown of complex substrates (Fig. 6). Although both *P. portentosus* and *Phlebopus* sp. exhibit dual lifestyles combining symbiosis and saprotrophy, the observed gene expansion and expression patterns suggest a stronger or more specialized saprotrophic capacity of *P. portentosus*.

Furthermore, analysis of secondary metabolite biosynthetic gene clusters (smBGCs) across 21 *Boletales* species revealed an expansion of NRPS and T1PKS pathways in *P. portentosus*, coupled with a reduction in terpenoid biosynthesis (Fig. 3). NRPS and T1PKS expansions likely enhance ecological competitiveness and stress resilience in soil environments by enabling the production of metabolites that inhibit soil microbiota or confer resistance to pathogen invasion (Challis and Hopwood 2003; Raaijmakers et al. 2016), while the loss of terpenoid pathways which typically associated with host interaction in ectomycorrhizal fungi reflects reduced dependence on symbiotic relationships (Ditengou et al. 2015; Plett et al. 2024). This also suggests a functional transition from symbiotic signaling toward independent survival strategies.

## ﻿Discussion

### ﻿Genetic and functional differences between two sexually compatible nuclei in *Phlebopus portentosus*

Recent advances in long-read sequencing have established T2T, haplotype-phased assemblies as the gold standard for eukaryotic genomics (Chen et al. 2024; Wang et al. 2024; Tam et al. 2025). In this context, the high-quality assemblies of sexually compatible *P. portentosus* strains PP78 and PP85 provided a robust foundation for downstream genomic and functional investigations. While strains PP17026 and PP33 exhibited lower assembly quality (Table 1), and another strain reported by Li (2020) achieved Hi-C scaffolding onto 11 chromosomes, the assembly still contained numerous internal gaps and a modest contig N50 of 1.20 Mb, with no publicly available data. The YAF023 strain assembly has been improved from 23 to 15 contigs (N50: 2.64 Mb), but remains below chromosome-level resolution and has a BUSCO completeness of 95.90% (Yang et al. 2024; Kang et al. 2025). In contrast, our PP78 and PP85 assemblies showed high mapping rates, contig N50 values of 2.62 Mb and 3.06 Mb, QV scores exceeding 55, and 99.60% BUSCO completeness, surpassing all previously reported *P. portentosus* genomes in continuity, completeness, and accuracy. These assemblies offer a valuable opportunity to explore genetic divergence between two compatible nuclei within a dikaryotic system.

Comparative studies of sexually compatible fungal strains have traditionally emphasized allelic variation, whereas large-scale structural differences have received comparatively little attention (Gao et al. 2022; Chen et al. 2024; Tam et al. 2025). The observed genome size difference of 1.17 Mb (Table 1) is primarily attributed to variation in repetitive element content, with PP85 harboring significantly more repetitive sequences, especially LTR retrotransposons of the *Copia* family (Table 2). These findings suggest that TE proliferation, particularly of *Copia* elements, is a major force driving genome plasticity and architectural diversification in *P. portentosus*. This is consistent with previous observations in other fungi, where TE bursts are linked to genome expansion, chromosomal restructuring, and the evolution of lineage-specific functions, potentially supporting adaptive responses to fluctuating environmental conditions (Castanera et al. 2016; Faino et al. 2016; Schrader and Schmitz 2019).

Despite a high degree of synteny between the two genomes, structural variations such as inversions, translocations, and segmental duplications were abundant (Fig. 4). The most prominent disparity lies in the expansion of PP85 Chr1, which is 1.41 Mb longer than its PP78 homolog and exhibits a markedly lower GC content (47.72% vs. 49.71%). This expansion is largely attributable to TE accumulation, especially LTR retrotransposons, which dominate a ~1 Mb region unique to PP85 Chr1 (Fig. 4D). The recent insertion times of these elements (*P* < 0.05, Suppl. material 2: fig. S10) indicate ongoing genome remodeling, echoing findings in other basidiomycetes where retrotransposons shape genome evolution (Castanera et al. 2016; Muszewska et al. 2019).

The rearrangements occurred not only between homologous chromosomes but also among non-homologous ones. A ~500 Kb fragment was transferred from PP78 Chr2 to non-homologous chromosome PP85 Chr3, likely resulted from a DNA_Harbinger-mediated “cut-and-paste” transposition (Fig. 4C). This suggested TE-mediated structural dynamics, a phenomenon previously reported in some fungi, such as *Verticillium dahliae* (Torres et al. 2021). Functional divergence was further evidenced by the identification of 187 PP78-specific and 236 PP85-specific protein-coding genes. Although most of these are annotated as hypothetical proteins, a notable subset in PP85 is enriched for TE-associated domains and predicted virulence factors (Suppl. material 1: table S12). In contrast, PP78-specific genes are predominantly associated with energy metabolism and chromosomal maintenance. This dichotomy may reflect a co-evolutionary strategy in dikaryons, balancing adaptability with stability, a phenomenon also observed in *L. edodes*, where mating-compatible strains exhibit compensatory genomic traits (Shen et al. 2024).

Although transposition activity was high at time 0 (Suppl. material 2: fig. S10), indicating ongoing transposon-mediated genome remodeling, the presence of PP85-specific regions in the genome of parental dikaryotic strain Ph5-2 rules out their origin during protoplast-based monokaryon isolation. Instead, these transposon-rich sequences were pre-existing structural components of the parental genome (Suppl. material 2: figs S11, S12).

While these findings underscore substantial structural and functional divergence between the two nuclei, the extent to which such nuclear asymmetry is conserved across the species remains unclear. Using Chr1 of strain PP85 as a reference, mapping resequencing data from additional dikaryotic strains (Ph7BN1-2, Ph46SM1-3, and Ph64WS2-2) (Suppl. material 2: fig. S12) confirmed that such inter-nuclear divergence is not exclusive to Ph5-2. However, determining its broader prevalence and biological significance will require further studies with more complete dikaryotic genome assemblies.

Large-scale chromosomal differences, particularly those driven by TE insertions, may interfere with homologous pairing during meiosis and suppress recombination, as documented in diverse eukaryotes. In organisms from *Drosophila* to plants, chromosomal rearrangements such as inversions and translocations have been shown to suppress crossover formation within and near rearranged regions (Kirkpatrick and Barton 2006; Faria and Navarro 2010). Structural heterozygosity may further suppress recombination *via* crossover interference, diminishing genetic exchange across entire chromosomes. Although evidence in fungi is limited, comparable phenomena have been observed in certain fungal hybrids, where extensive structural divergence correlates with reduced recombination and fertility (Stukenbrock and Croll 2014; Chuang et al. 2015). This raises the possibility that TE-driven structural divergence in this species could influence reproductive compatibility, a hypothesis that merits testing through broader sampling of dikaryotic isolates and cytogenetic analyses.

Transcriptomic analysis showed that strain-specific genes, although generally expressed at lower levels than core genes (Suppl. material 2: fig. S13B–D), are transcriptionally active. Their developmental stage-specific expression suggests potential roles in environmental responsiveness or morphogenetic transitions. Notably, the orsellinic acid-like gene cluster found exclusively in PP85, though transcriptionally silent under tested conditions, may constitute a cryptic secondary metabolic pathway inducible by specific environmental stimuli (Lim et al. 2012).

Altogether, the genetic divergence between the two nuclei in *P. portentosus* reveals a nuanced balance between genomic stability and flexibility, likely reflecting a co-evolutionary adaptation within dikaryotic systems. This nuclear heterogeneity may confer both resilience and versatility, as similarly observed in *L. edodes* (Gao et al. 2022) and *Agaricus bisporus* (Gehrmann et al. 2018), where nuclear dimorphism contributes unequally to traits linked to fitness and ecological performance. These insights enhance our understanding of how nuclear differentiation drives functional and evolutionary diversification in basidiomycetes.

### ﻿Transposon-driven structural variations shape secondary metabolite gene clusters in *Phlebopus portentosus*

In *P. portentosus*, genomic comparisons between strains PP85 and PP78 highlighted the central role of TEs in driving both structural divergence and functional diversification. These differences were especially pronounced in not only chromosome size variation, but also the organization of smBGCs, suggesting a close link between TE activity and genome architecture dynamics.

As noted above, a ~1 Mb TE-rich region unique to PP85 Chr1 is highly enriched in LTR retrotransposons, indicating ongoing retrotransposon activity that has contributed to the genomic divergence between PP85 and PP78. This observation reinforces the broader paradigm that TEs are key drivers of both large-scale genome expansion and fine-scale structural variation in closely related fungal lineages (Daboussi and Capy 2003; Schrader and Schmitz 2019).

Comparative analyses of smBGCs revealed distinct organizational patterns between PP78 and PP85, including TE-associated splitting, merging, and relocation events. DNA transposons, particularly those employing a “cut-and-paste” mechanism, appeared to mediate several of these rearrangements, further implicating mobile elements in the evolutionary reconfiguration of fungal secondary metabolism.

Beyond structural impact, TEs also appear to influence the transcriptional activity of smBGCs. Insertions near regulatory regions were associated with altered expression profiles of key biosynthetic genes in PP85 relative to PP78, suggesting that the proximity of transposons can modulate gene expression, a phenomenon previously demonstrated in other fungi (Khaldi et al. 2010; Lind et al. 2017).

The structural and transcriptional variation of smBGCs between *P. portentosus* strains highlights transposon-mediated genome plasticity as a potent driver of metabolic diversity. This dynamic feature offers valuable insights for strain improvement and may serve as a strategic foundation for future efforts in fungal domestication and biotechnological innovation.

### ﻿Comparative genomic analysis revealed features associated with the saprotrophic capacity of *P. portentosus*

Nutritional strategies are closely linked to fungal evolution and ecological adaptation (Gladieux et al. 2014; Stajich 2017; Naranjo-Ortiz and Gabaldón 2019; Martin et al. 2024), and are also one of the key factors influencing the successful domestication and artificial cultivation of wild edible fungi (Lu et al. 2020; Pérez-Moreno et al. 2021). Comparative genomic studies have shown that ECM fungi, in contrast to saprotrophs, exhibited marked genomic modifications, including reductions in carbohydrate-active enzymes (CAZymes), loss of plant cell wall degrading enzymes (PCWDs), expansion of small secreted proteins (SSPs), and proliferation of TEs (Van et al. 2015; Miyauchi et al. 2020). Genomic analysis of *P. portentosus* revealed a CAZyme repertoire similar to that of ECM fungi, characterized by the absence of key lignocellulose-degrading genes and PCWDs, along with an abundance of TEs. These features supported its classification as an ECM fungus (Li 2020; Wan et al. 2021), consistent with the symbiotrophic signature previously reported for *P. portentosus* (Wu et al. 2022).

However, our comparative genomic analysis uncovered several genomic features associated with the saprotrophic capacity of *P. portentosus*: (1) a higher abundance of GH31 glycoside hydrolases, a large and functionally diverse family encompassing glycosidases, lyases, and transglycosidases and α-xylosidase, which are involved in the efficient assimilation of carbohydrates during substrate colonization; (2) the significant expansion and active transcription of gene families encoding acetyl-CoA synthetase-like proteins, peroxidases, and sugar transporters involved in the degradation of complex substrates; and (3) expansion of NRPS and T1PKS pathways (likely enhances ecological competitiveness and stress resilience in soil environments) and a reduction in gene clusters related to terpenoid biosynthesis (typically associated with host interactions in ectomycorrhizal fungi) (Ditengou et al. 2015; Plett et al. 2024).

The most recent common ancestor of *Boletales* was presumed to have been a brown-rot fungus (Ruiz-Dueñas et al. 2020). We proposed that *P. portentosus* represents an intermediate form that has not yet fully transitioned to a symbiotrophic lifestyle. These retained saprotrophic traits have likely contributed to the successful large-scale artificial cultivation of *P. portentosus*. Building on this perspective, the domestication of additional Boletus species may be achievable in the future.

## ﻿Conclusions

Our study provides the T2T, chromosome-level nuclear genomes of two sexually compatible monokaryons of *P. portentosus*, offering critical insights into its genome evolution and facultative ectomycorrhizal lifestyle. These assemblies represent the highest quality genomes reported for this species to date. TEs emerged as major drivers of genome plasticity, nuclear differentiation, and metabolic diversity, shaping structural and functional divergence between the two compatible nuclei. Comparative analysis revealed genomic traits consistent with a facultative lifestyle, bridging ECM and free-living modes of nutrition. These high-quality genomic resources lay a valuable foundation for future research on the molecular mechanisms underlying trophic flexibility, secondary metabolism, and cultivation improvement in *P. portentosus*.

## ﻿Additional information

### Use of AI

No use of AI was reported.

### Adherence to national and international regulations

All the fungal strains used in this study have been legally obtained, respecting the Convention on Biological Diversity (Rio Convention).

### Author ORCIDs

Guoliang Meng https://orcid.org/0009-0008-5217-0753

Jiajia Li https://orcid.org/0009-0006-7383-6791

Yao Cao https://orcid.org/0000-0002-8421-4094

Fan Li https://orcid.org/0009-0002-9069-508X

MengQian Liu https://orcid.org/0009-0004-4758-1903

Rongchun Li https://orcid.org/0000-0001-5728-6618

Caihong Dong https://orcid.org/0000-0002-2558-3404

### Data availability

All raw sequencing data generated in this study have been deposited in the NCBI BioProject database (https://www.ncbi.nlm.nih.gov/bioproject/). HiFi reads for strains PP78 and PP85, as well as MGISEQ-T7 resequencing data for the dikaryotic strains Ph5-2, Ph7BN1-2, Ph46SM1-3, and Ph64WS2-2, are available under accession number PRJNA1269926.

## Supplementary materials

Supplementary material 1 Supplementary tables S1–S16 Data type xlsx https://binary.pensoft.net/file/1404105This dataset is made available under the Open Database License (http://opendatacommons.org/licenses/odbl/1.0/). The Open Database License (ODbL) is a license agreement intended to allow users to freely share, modify, and use this Dataset while maintaining this same freedom for others, provided that the original source and author(s) are credited.Guoliang Meng, Jiajia Li, Yao Cao, Fan Li, MengQian Liu, Rongchun Li, Caihong Dong

Supplementary material 2 Supplementary figures S1–S17 Data type docx https://binary.pensoft.net/file/1404106This dataset is made available under the Open Database License (http://opendatacommons.org/licenses/odbl/1.0/). The Open Database License (ODbL) is a license agreement intended to allow users to freely share, modify, and use this Dataset while maintaining this same freedom for others, provided that the original source and author(s) are credited.Guoliang Meng, Jiajia Li, Yao Cao, Fan Li, MengQian Liu, Rongchun Li, Caihong Dong
