# Supplementary material for: ﻿Haplotype-resolved genomes of Phlebopus portentosus reveal nuclear differentiation, TE-mediated variation, and saprotrophic potential
Source: IMA Fungus. 2025 Aug 28;16:e161411. doi: 10.3897/imafungus.16.161411 (PMC12411881; doi:10.3897/imafungus.16.161411)
Supplement: Supplementary material 2 — Supplementary figures S1–S17 [file imafungus-16-e161411-s002.docx]

**Supplementary materials**

**Manuscript title:** **Haplotype-resolved genomes of *Phlebopus portentosus* reveal nuclear differentiation, TE-mediated variation, and saprotrophic potential**

**Guoliang Meng^1^, Jiajia Li^1^, Yao Cao^2^, Fan Li^2^, MengQian Liu^1^, Rongchun Li^2*^ and Caihong Dong^1*^**

1 State Key Laboratory of Microbial Diversity and Innovative Utilization, Institute of Microbiology, Chinese Academy of Sciences, Beijing, 100101, China

2 Yunnan Junshijie Biotechnology Ltd., Kunming 650200, Yunnan, China

*Corresponding authors. E-mail addresses: dongch@im.ac.cn (C. Dong), rongchunli@126.com (R. Li)

**Information**

**Supplementary Figure S1 to Figure S17.**

**Figure S1.**


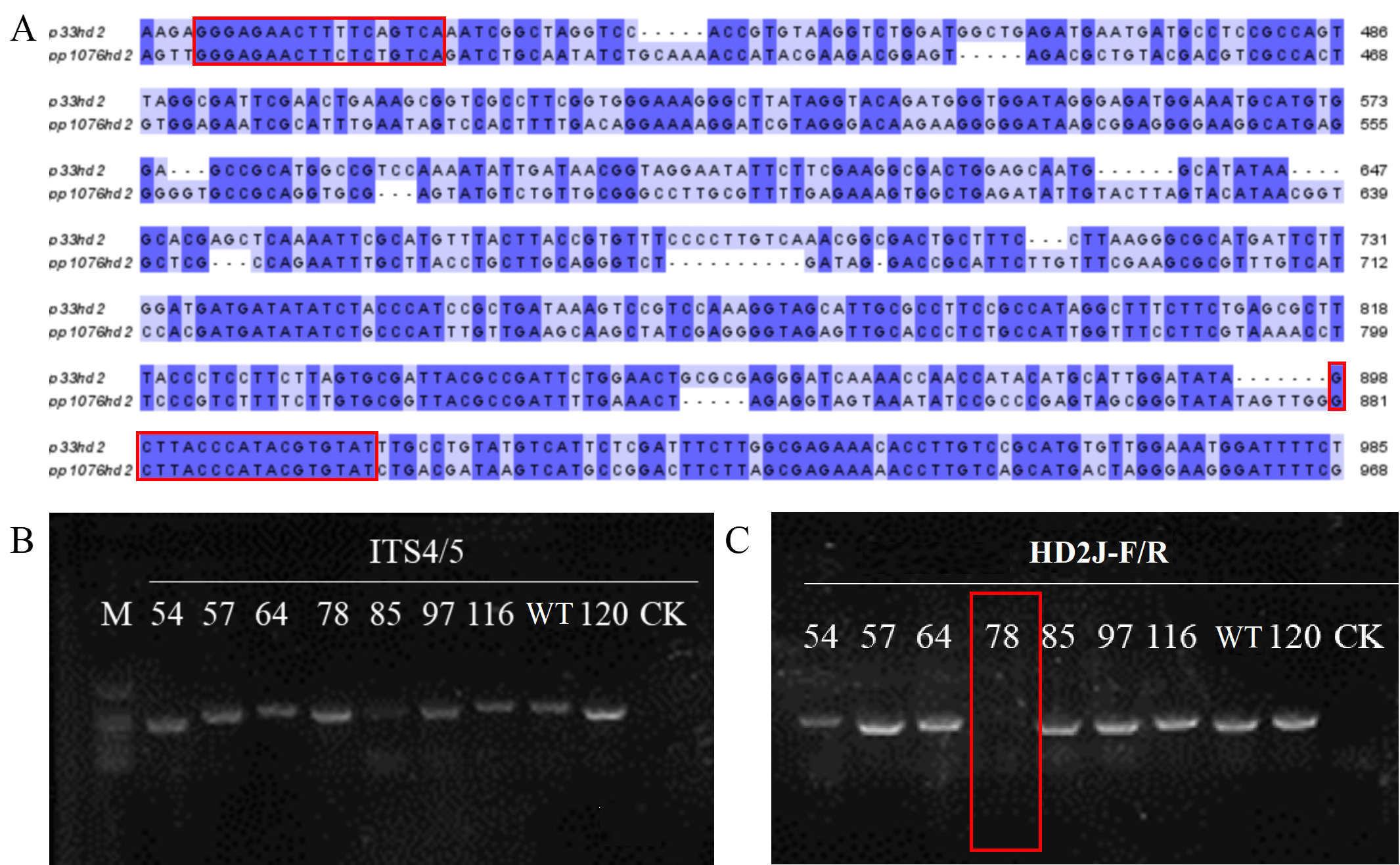


**Figure S1.** Verification of sexually compatible strains of *Phlebopus portentosus* based on mating-type-specific primers with amplification **A** Based on the alignment results of the HD2 gene from the available genomes, the red box indicates the selected degenerate primers. **B** ITS and HD2 amplification of putative monokaryotic strains. M indicates 2K Marker, 1-9 are the monokaryotic strains, 10 indicates the wild type strain, H_2_O indicates the blank controls.

**Figure S2.**


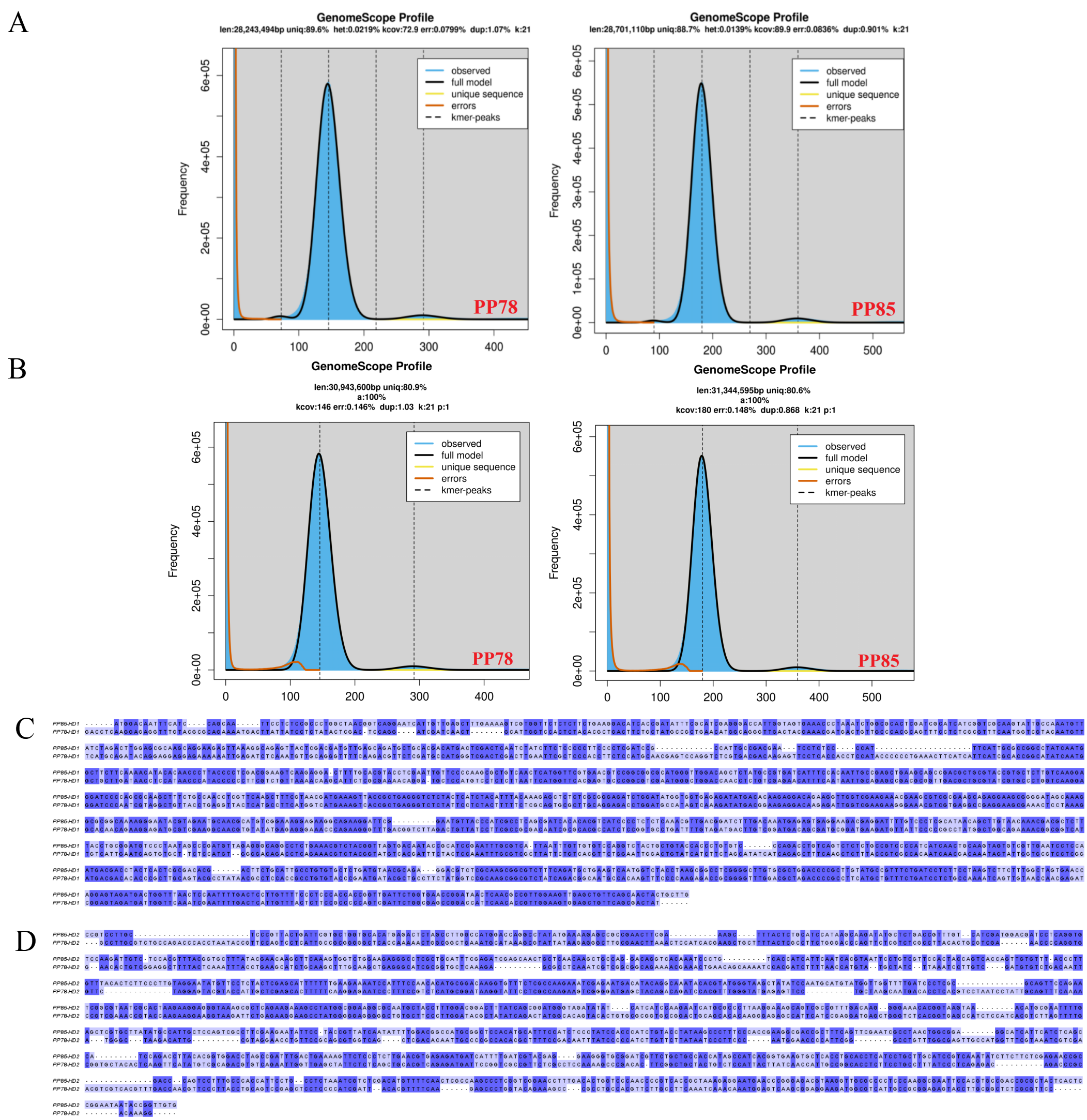


**Figure S2.** Verification of sexually compatible strains of *Phlebopus portentosus* through heterozygosity analysis and mating-type gene comparison **A** The GenomeScope profiles of PP78 and PP85 based on next-generation sequencing with 21 K-mer with ploidy setting of 2. **B** The GenomeScope profiles of PP78 and PP85 based on next-generation sequencing with 21 K-mer with ploidy setting of 1. **C** PP78 and PP85 mating-type gene *HD*1 comparison. **D** PP78 and PP85 mating-type gene *HD*2 comparison.

**Figure S3.**


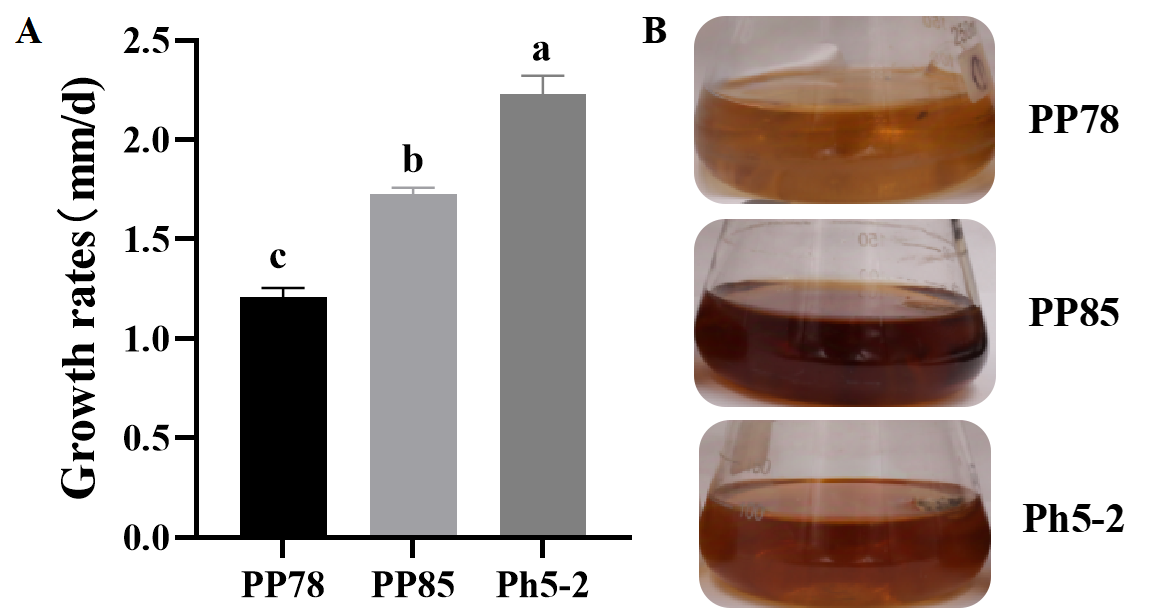


**Figure S3.** Growth rate and phenotypic observation of *Phlebopus portentosus* strains. **A** Growth rate of *Phlebopus portentosus* strains. **B** Pigment variation of *Phlebopus portentosus* strains under identical liquid culture durations.

**Figure S4.**


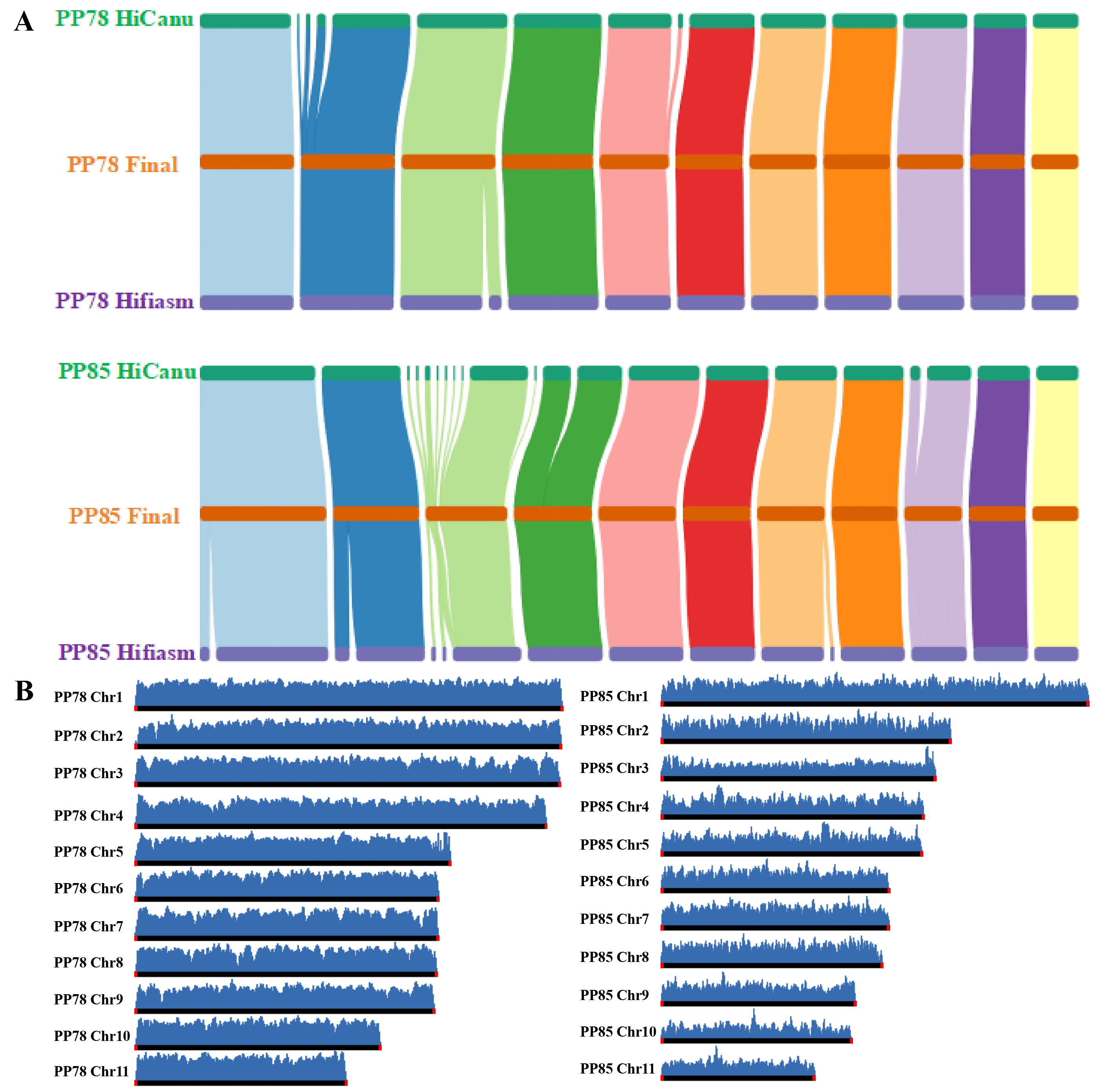


**Figure S4.** Genome Assembly of *Phlebopus portentosus*. **A** Comparison of draft genome assemblies from different software with the final version. **B** HiFi reads coverage of the 11 chromosomes of PP78 and PP85.

**Figure S5.**

**
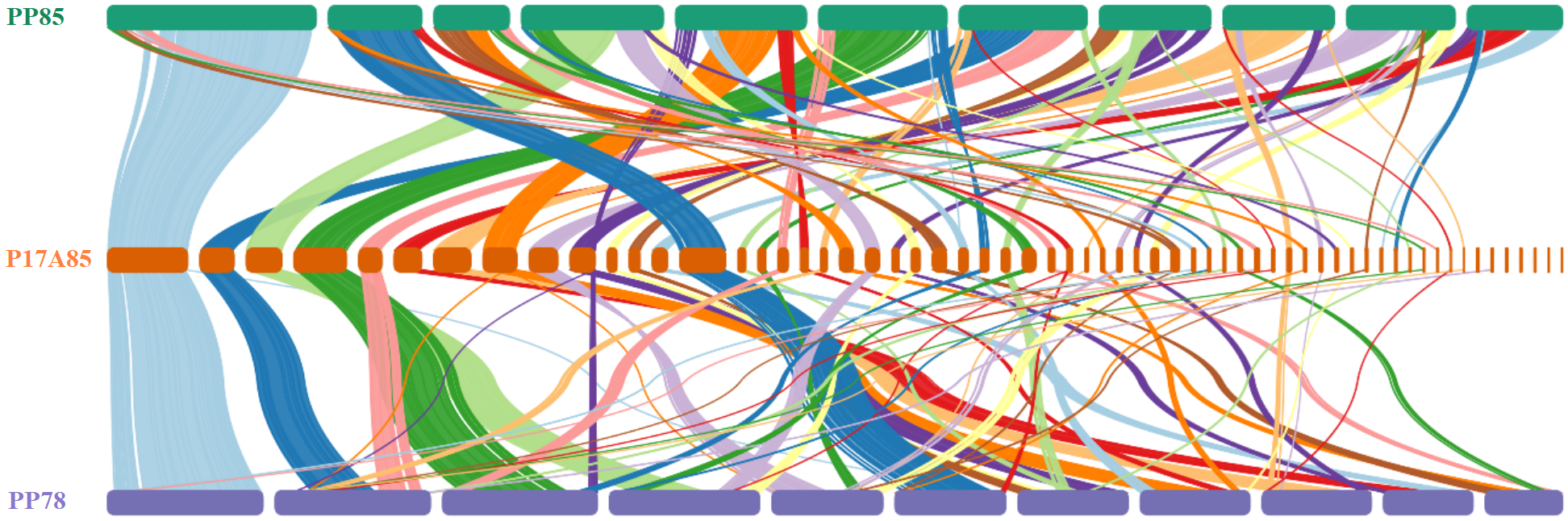
**

**Figure S5.** Genomic collinearity analysis of *Phlebopus portentosus*.

**Figure S6.**


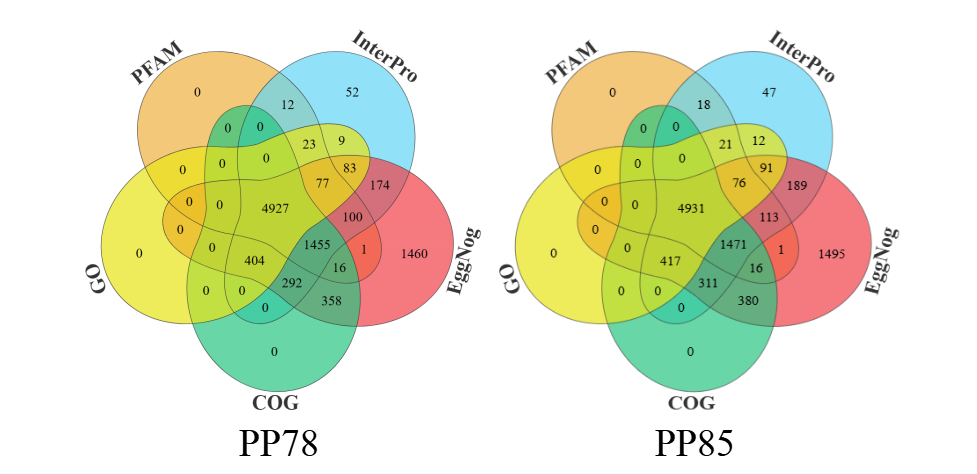


**Figure S6.** Genome protein function annotation of PP78 and PP85 by different method.

**Figure S7.**


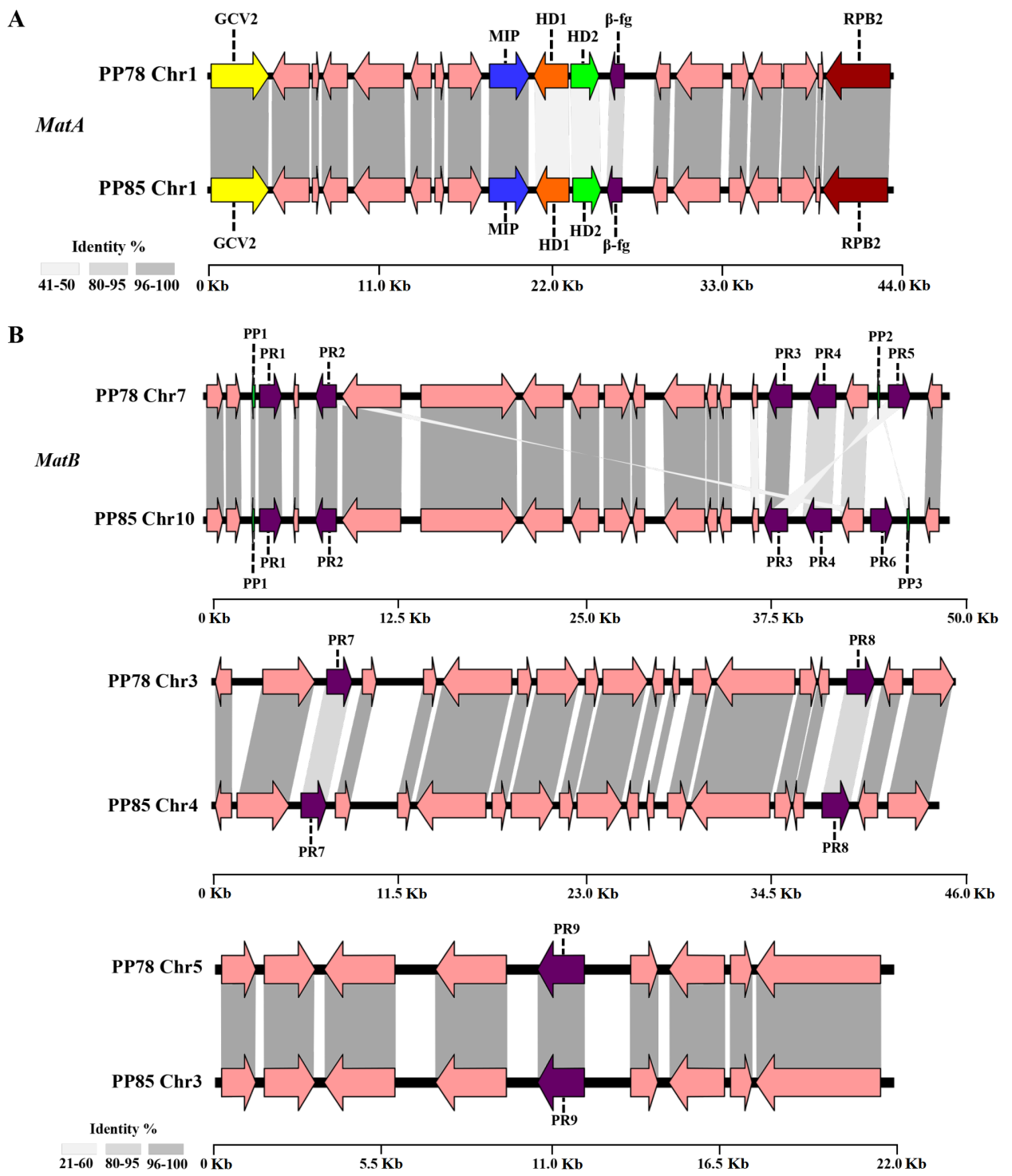


**Figure S7**. The protein synteny around the *MatA* and *MatB* loci of PP78 and PP85 **A** *MatA* loci of PP78 and PP85. HD: Homeodomain, MIP: mitochondrial intermediate peptidase, GCV2: glycine decarboxylase subunit P, RPB2: RNA polymerase II, the second large subunit, β-fg: *β-flanking*. **B** *MatB loci* of PP78 and PP85. PR: pheromone receptor, PP: pheromone precursor.

**Figure S8**.


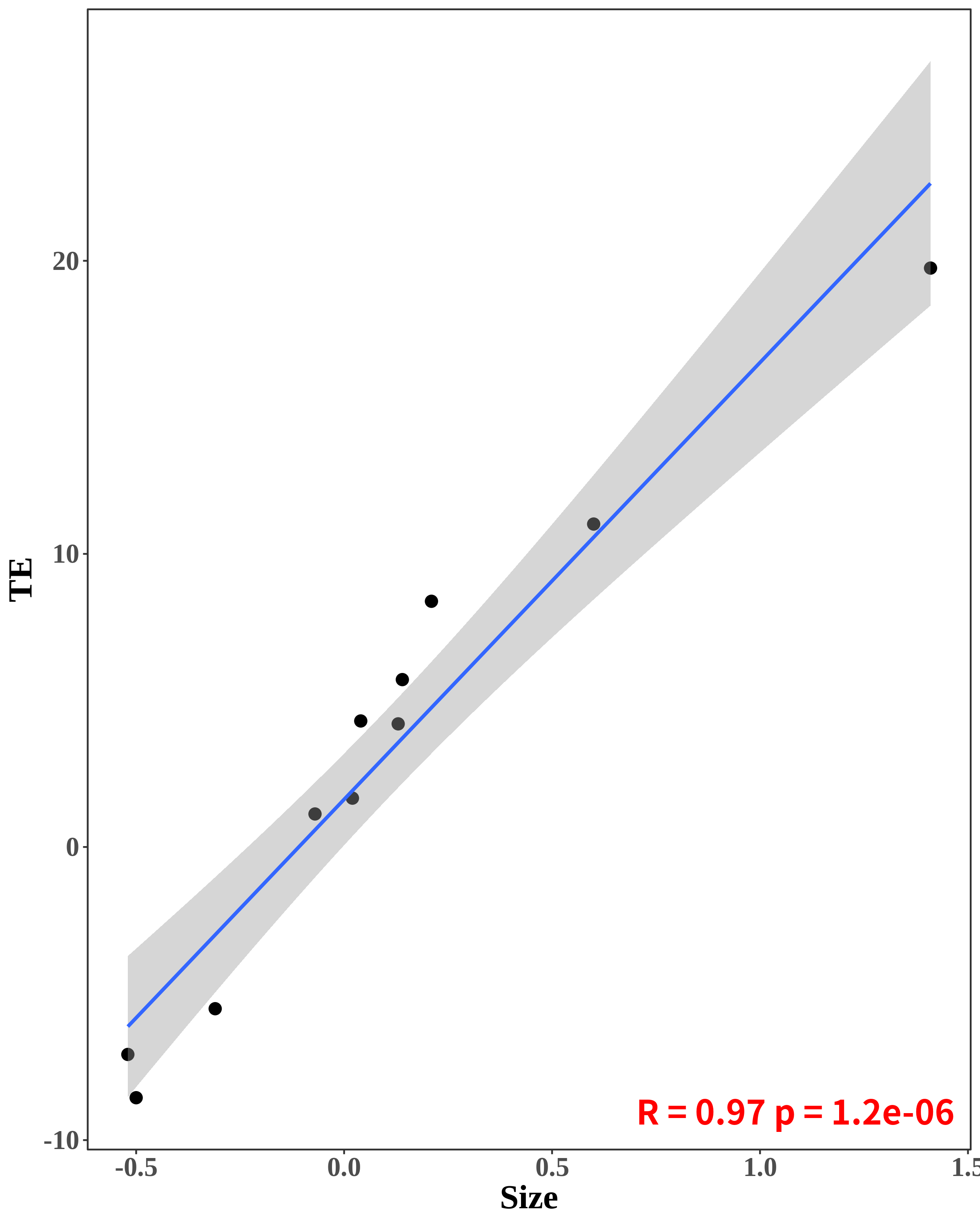


**Figure S8**. Comparative analysis of chromosomes size and TE contents variations in sexually compatible strains PP78 and PP85 of *Phlebopus portentosus*.

**Figure S9**.


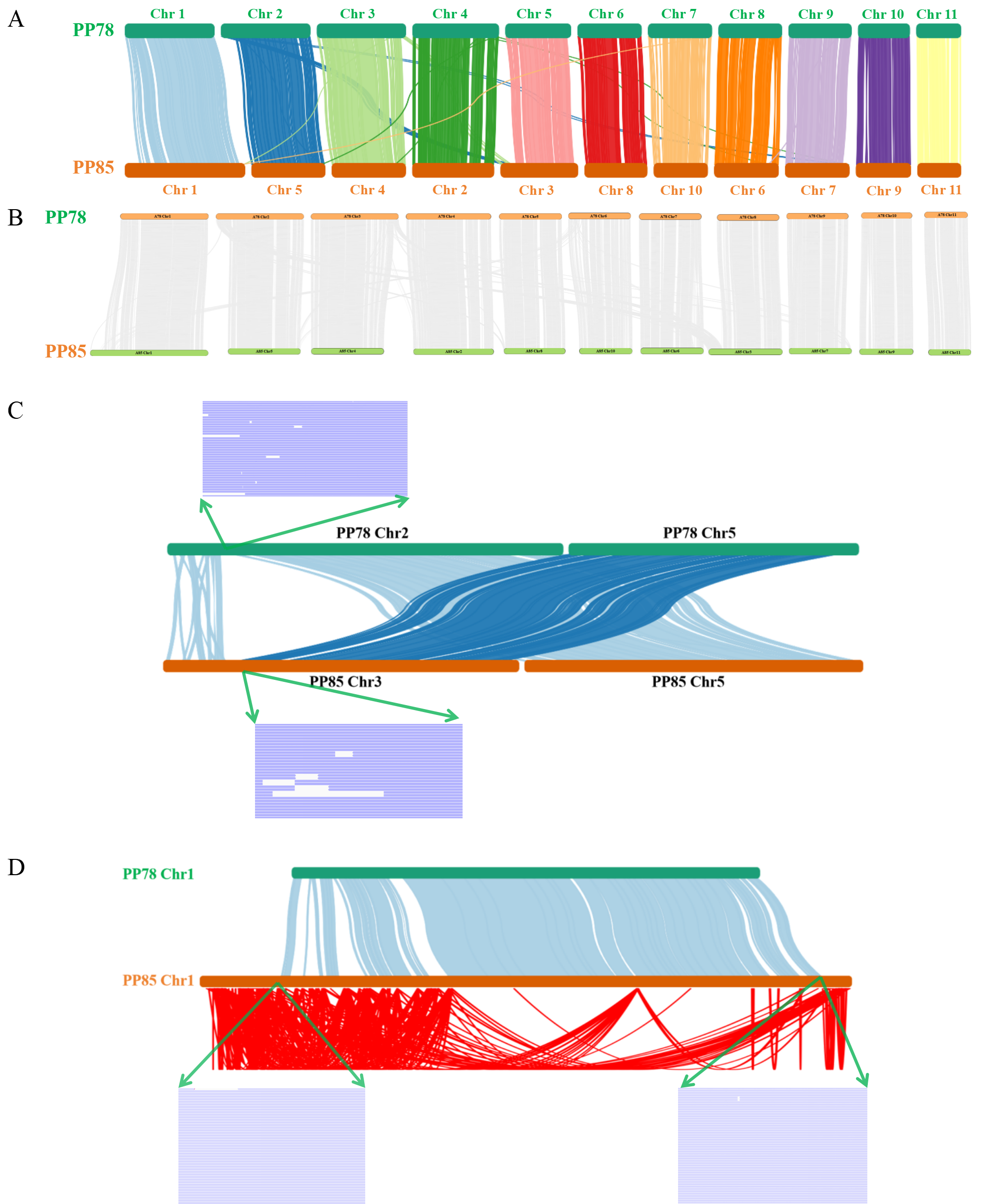


**Figure S9**. Comparative analysis of genome synteny and structural variations in *Phlebopus portentosus* **A** Synteny map based on nucleotide sequences. **B** Synteny map based on protein sequences.

**Figure S10**.


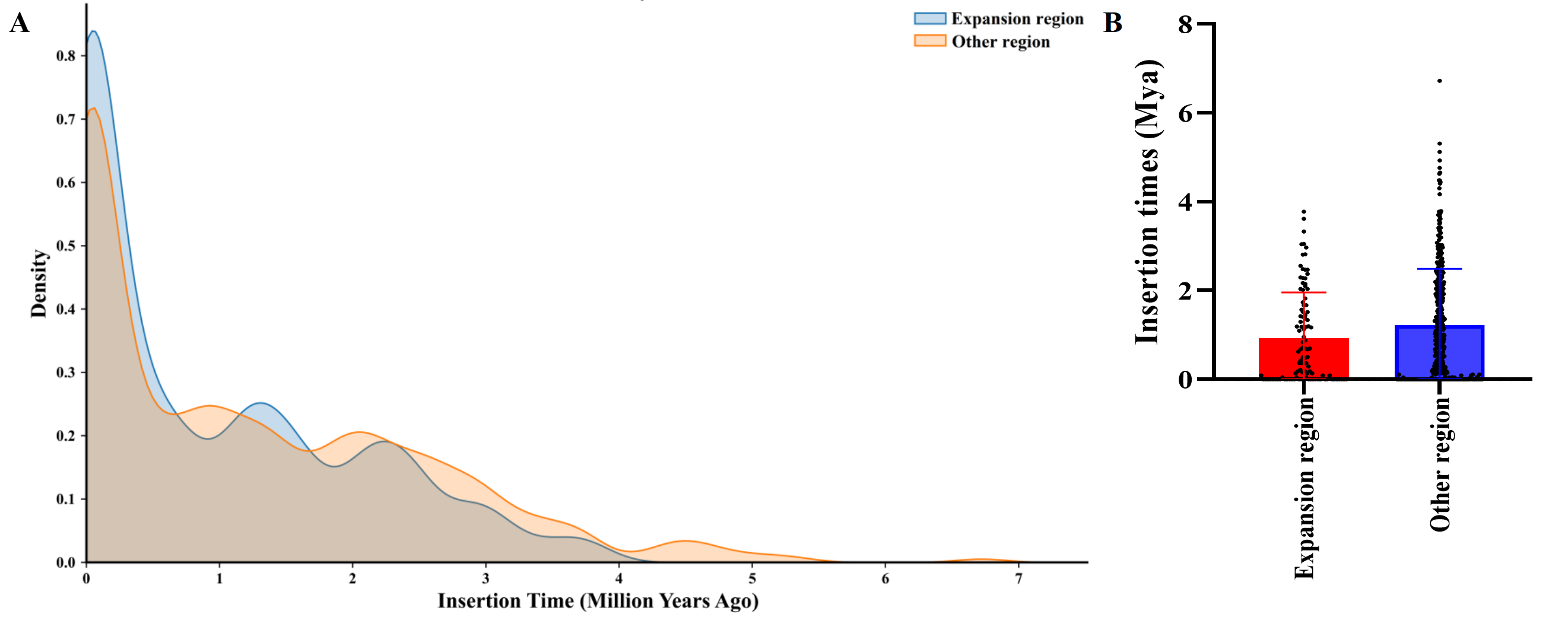


**Figure S10**. Transposon insertion time analysis in different genomic regions of the PP85 strain **A** Insertion time density distribution in PP85 Chr1 expansion region and other regions. **B** Insertion time in PP85 Chr1 expansion region and other regions.

**Figure S11**.


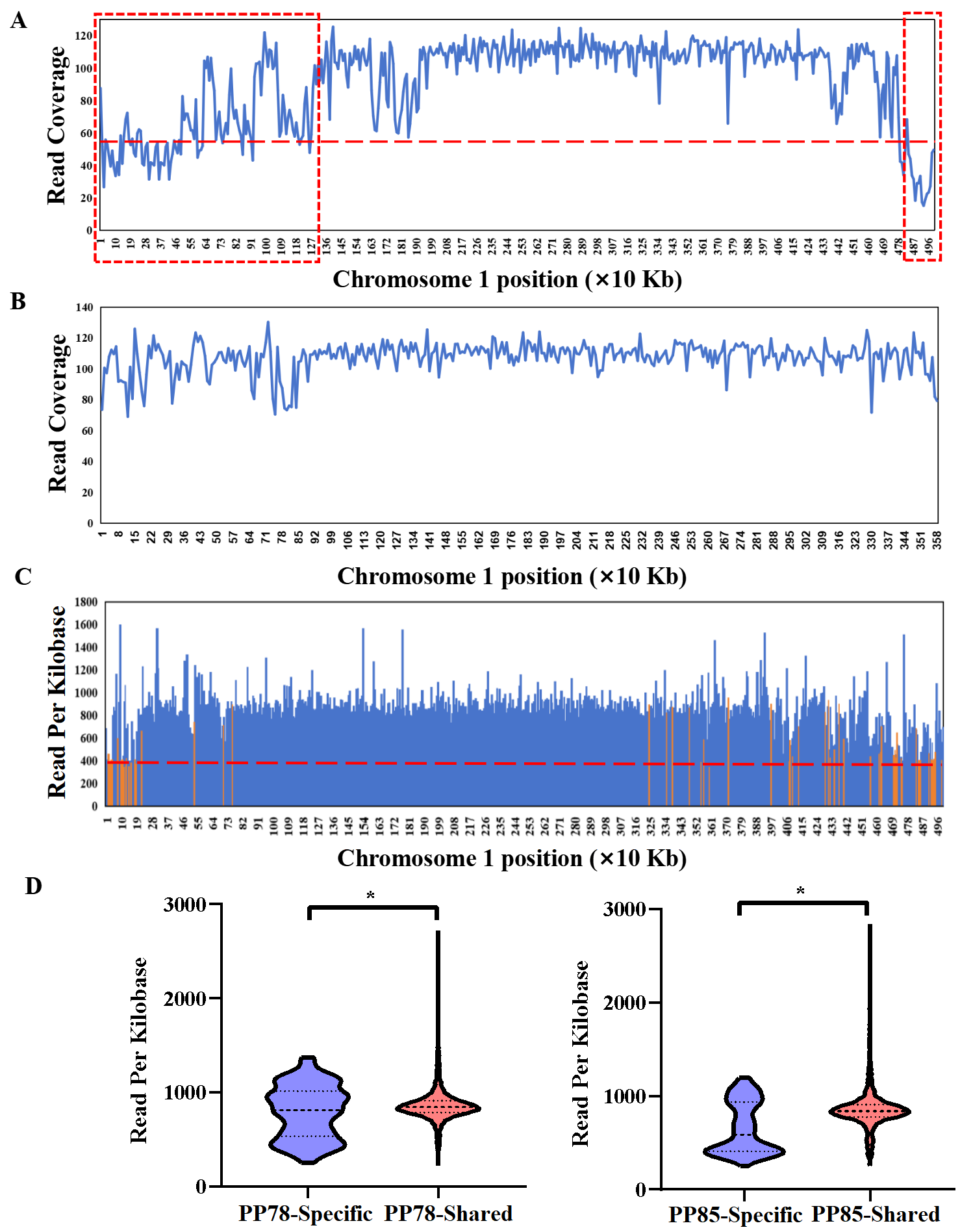


**Figure S11**. Read-depth profiles of Chr1 and reads per kilobase of genes using resequencing data from the parental dikaryotic strain Ph5-2. **A** Read-depth profile of Chr1 from monokaryotic strain PP85 mapped to Ph5-2 resequencing data. Red dashed lines indicate half the coverage of shared genomic regions; Red boxes mark PP85-specific regions. **B** Read-depth profile of Chr1 from monokaryotic strain PP78 mapped to Ph5-2 resequencing data. **C** Read per kilobase values for nucleus-specific (orange) and shared genes (blue) in PP85 Chr1. **D** Distribution of read per kilobase values for nucleus-specific and shared genes in PP78 and PP85.

**Figure S12**.


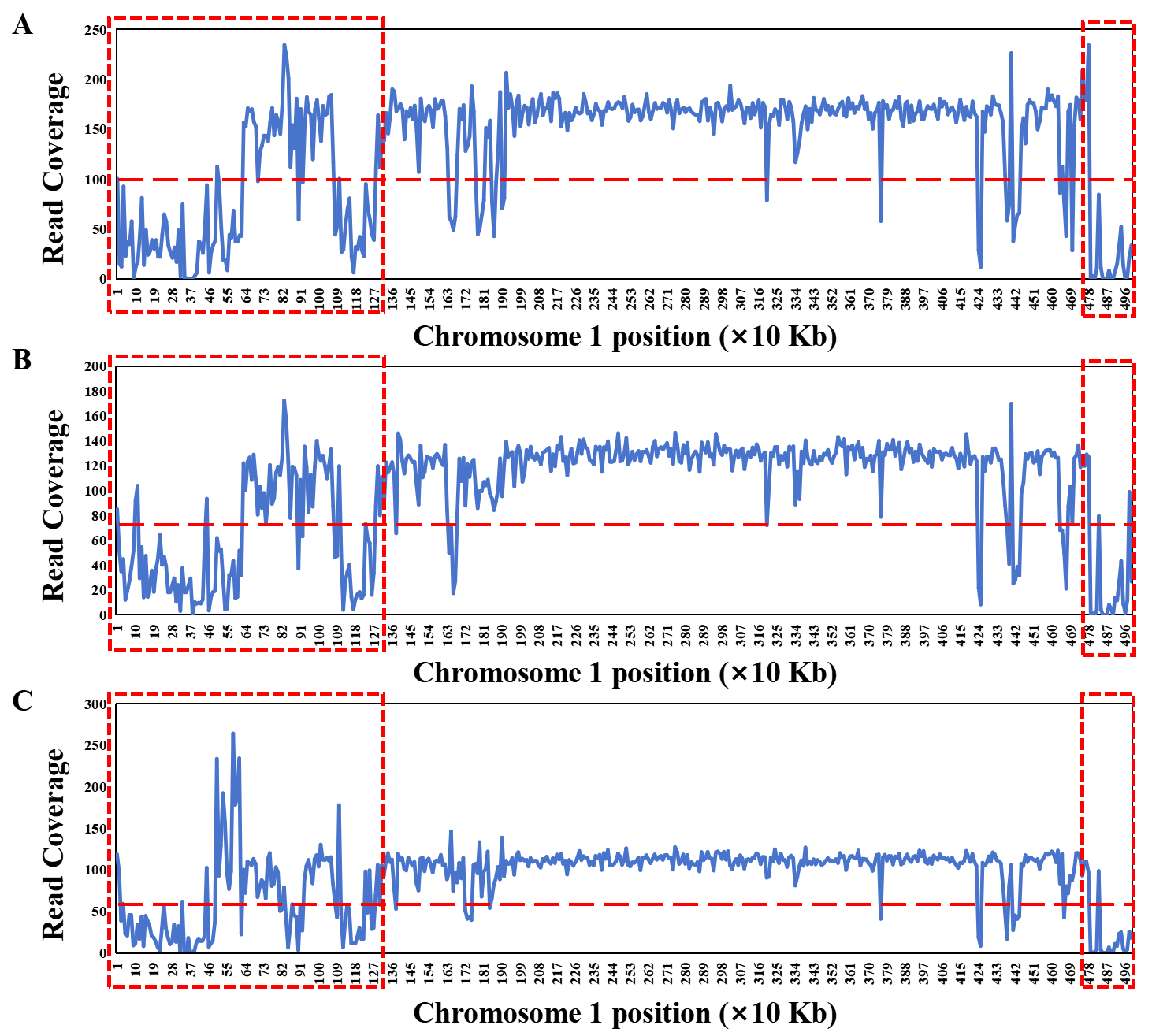


**Figure S12.** Read-depth profiles of Chr1 from monokaryotic strain PP85 mapped to resequencing data of various dikaryotic strains. **A** Mapped to Ph7BN1-2 resequencing data. **B** Mapped to Ph46SM1-3 resequencing data. **C** Mapped to Ph64WS2-2 resequencing data. Red dashed lines indicate half the read coverage. Red boxes highlight PP85-specific genomic regions.

**Figure S13**.


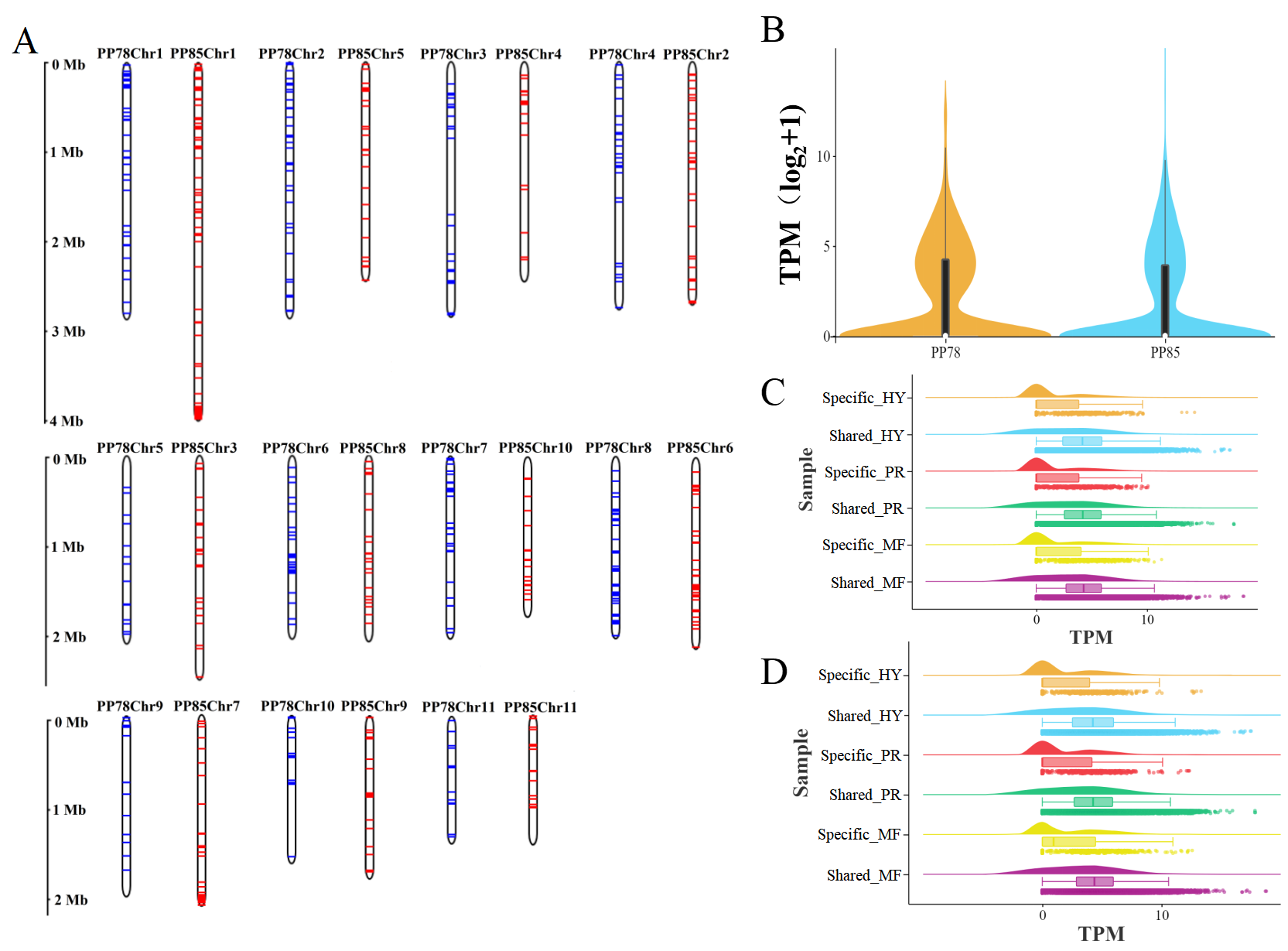


**Figure S13**. Distribution of strain-specific genes and differential gene expression analysis in sexually compatible strains. **A** Identification and distribution of strain-specific genes across sexually compatible strains. **B** Genome-wide expression analysis of lineage-specific genes in *Phlebopus portentosus* strains PP78 and PP85. **C** Expression analysis of PP78-specific genes and shared genes across developmental stages. **D** Expression analysis of PP78-specific genes and shared genes across developmental stages. **E** DNA and transcriptional validation of the Orsellinic acid-like gene. HY: hypha; PR: primordium; FB: fruiting bodies; M: marker.

**Figure S14**.


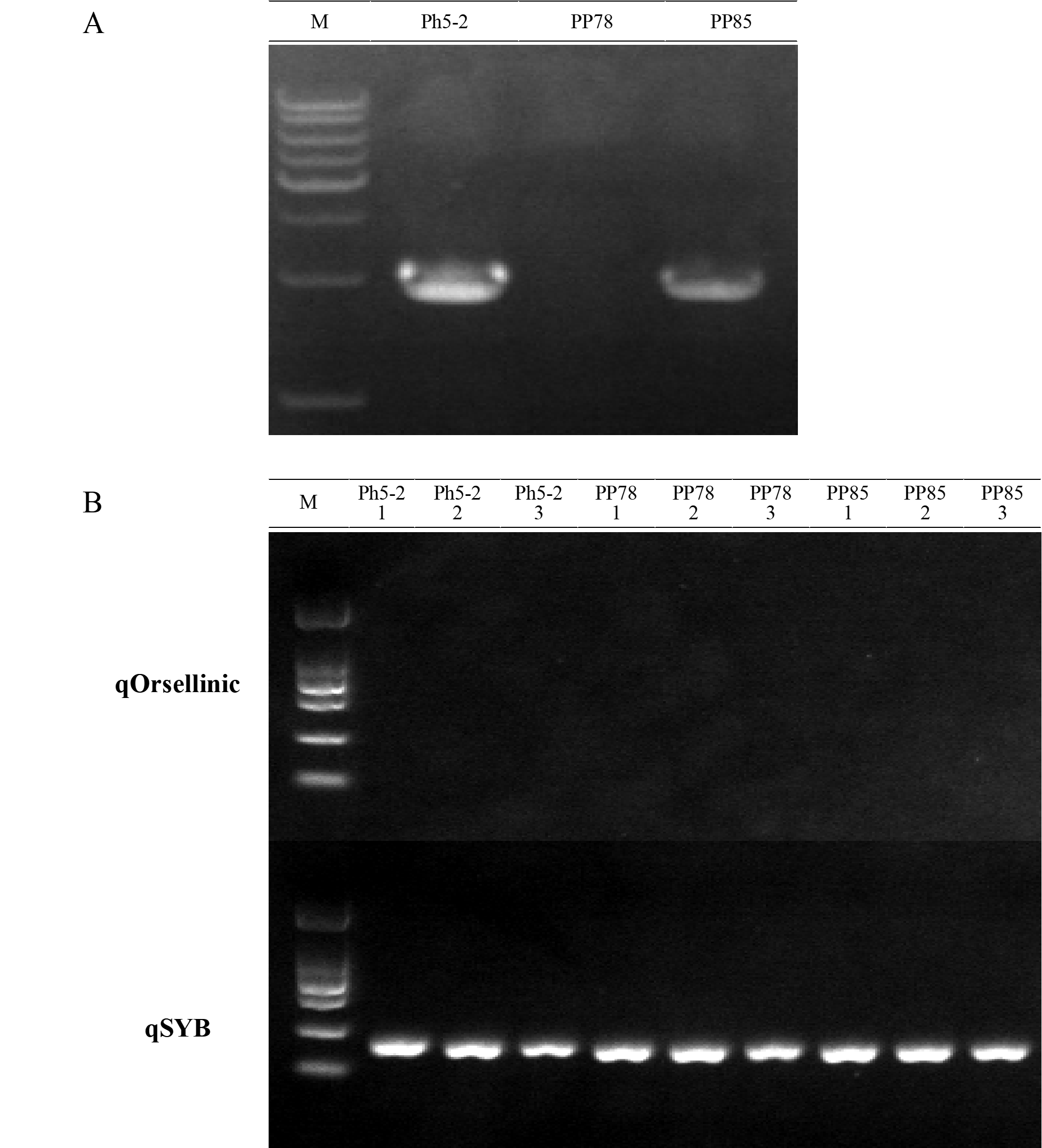


**Figure S14**. Validation of the authenticity of PP85-specific genes at stages of mycelium **A** DNA validation of the Orsellinic acid-like gene; **B** Transcriptional validation of the Orsellinic acid-like gene.

**Figure S15**.


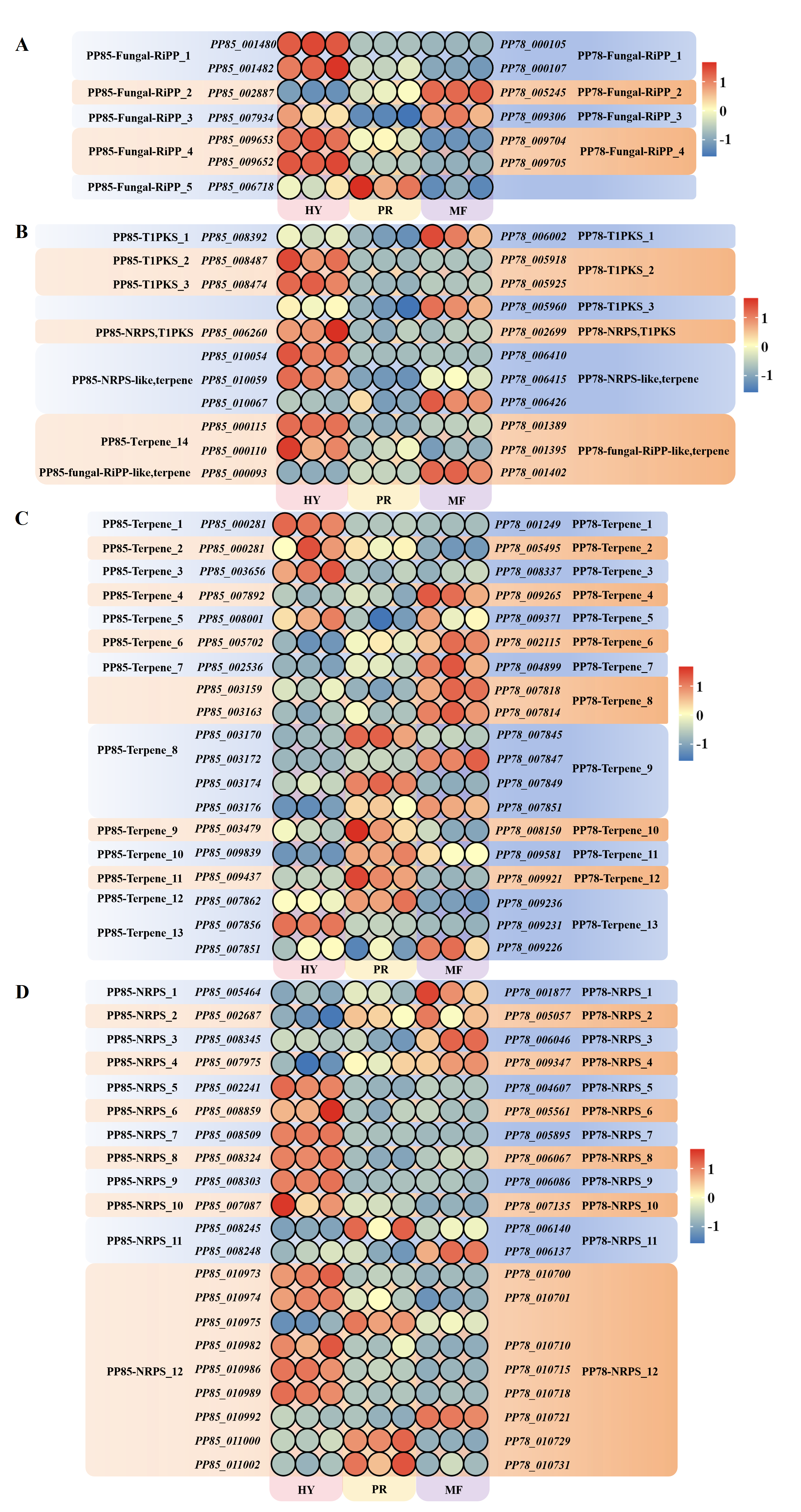


**Figure S15**. Expression analysis of core genes in secondary metabolite biosynthetic gene clusters of sexually compatible *Phlebopus portentosus* strains. HY: hypha; PR: primordium; FB: fruiting bodies.

**Figure S16**.


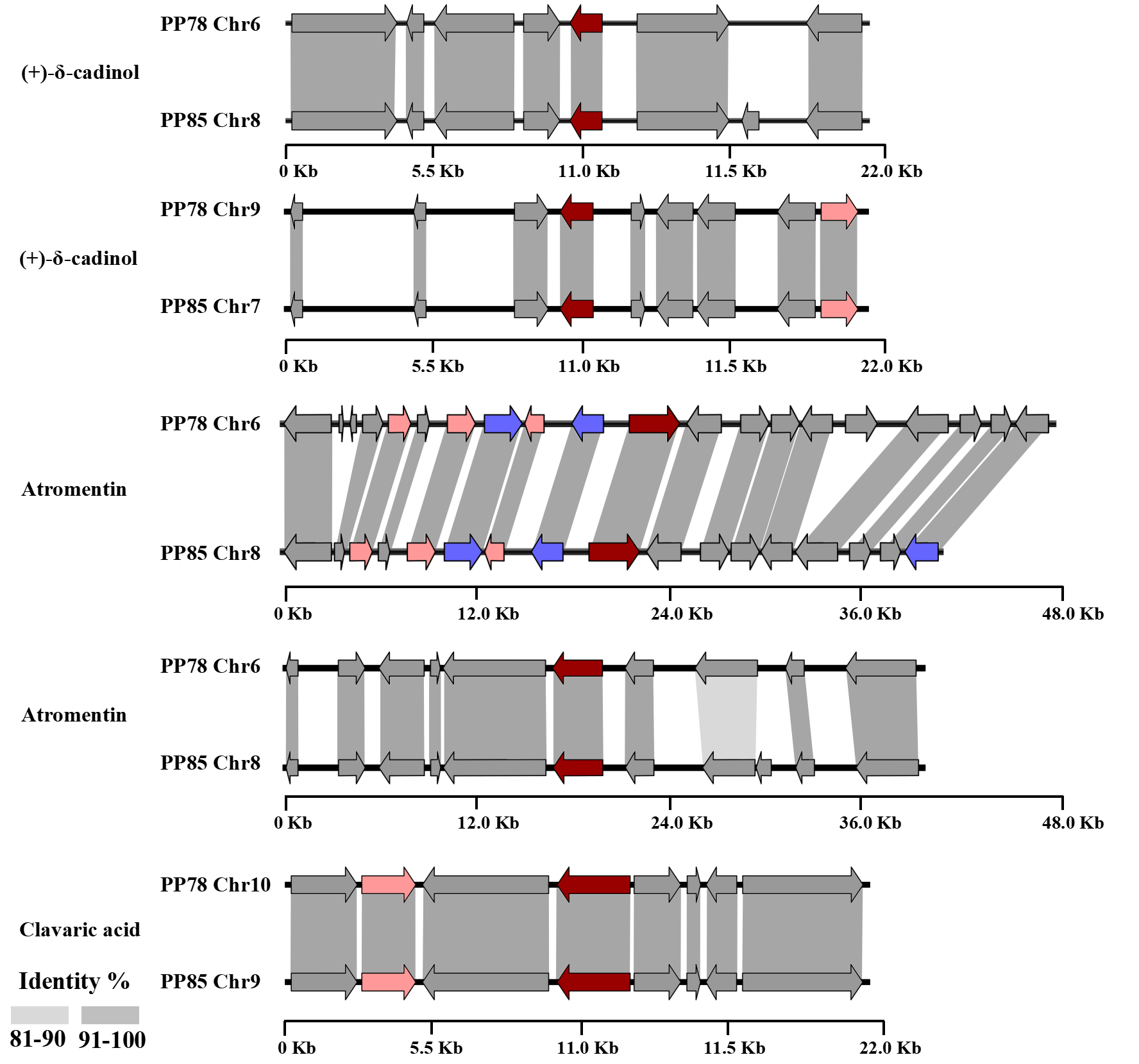


**Figure S16**. Three type secondary metabolite biosynthetic gene clusters linked to known bioactive metabolites of sexually compatible *Phlebopus portentosus* strains.

**Figure S17**.


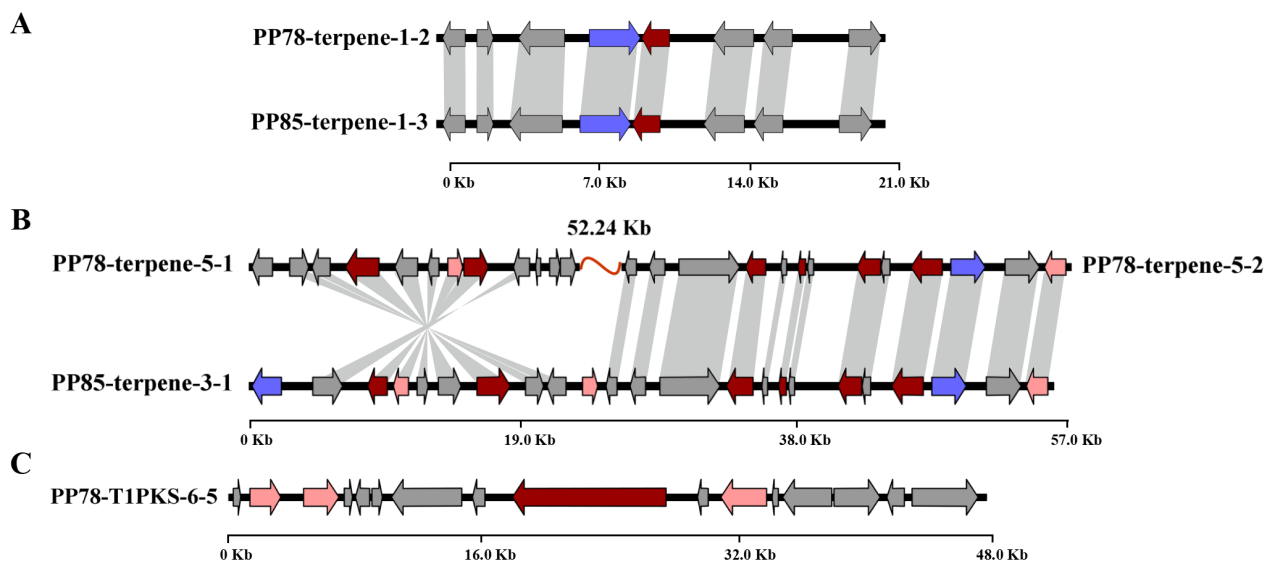


**Figure S17**. Comparative analysis of secondary metabolite biosynthetic gene clusters in the genomes of sexually compatible *Phlebopus portentosus* strains. **A** Highly conserved secondary metabolite biosynthetic gene clusters. **B** A terpene biosynthetic gene cluster in strain PP85 corresponds to two distinct terpene clusters in strain PP78. **C** Secondary metabolite biosynthetic gene clusters unique to strain PP78. Blue indicates transporter-related genes, pink represents additional biosynthetic genes, and brown denotes core biosynthetic genes.

**Figure S18**.

**Figure S18**. Comparative analysis of secondary metabolite biosynthetic gene clusters’ gene expression level in the genomes of sexually compatible *Phlebopus portentosus* strains.

**Figure S19**.


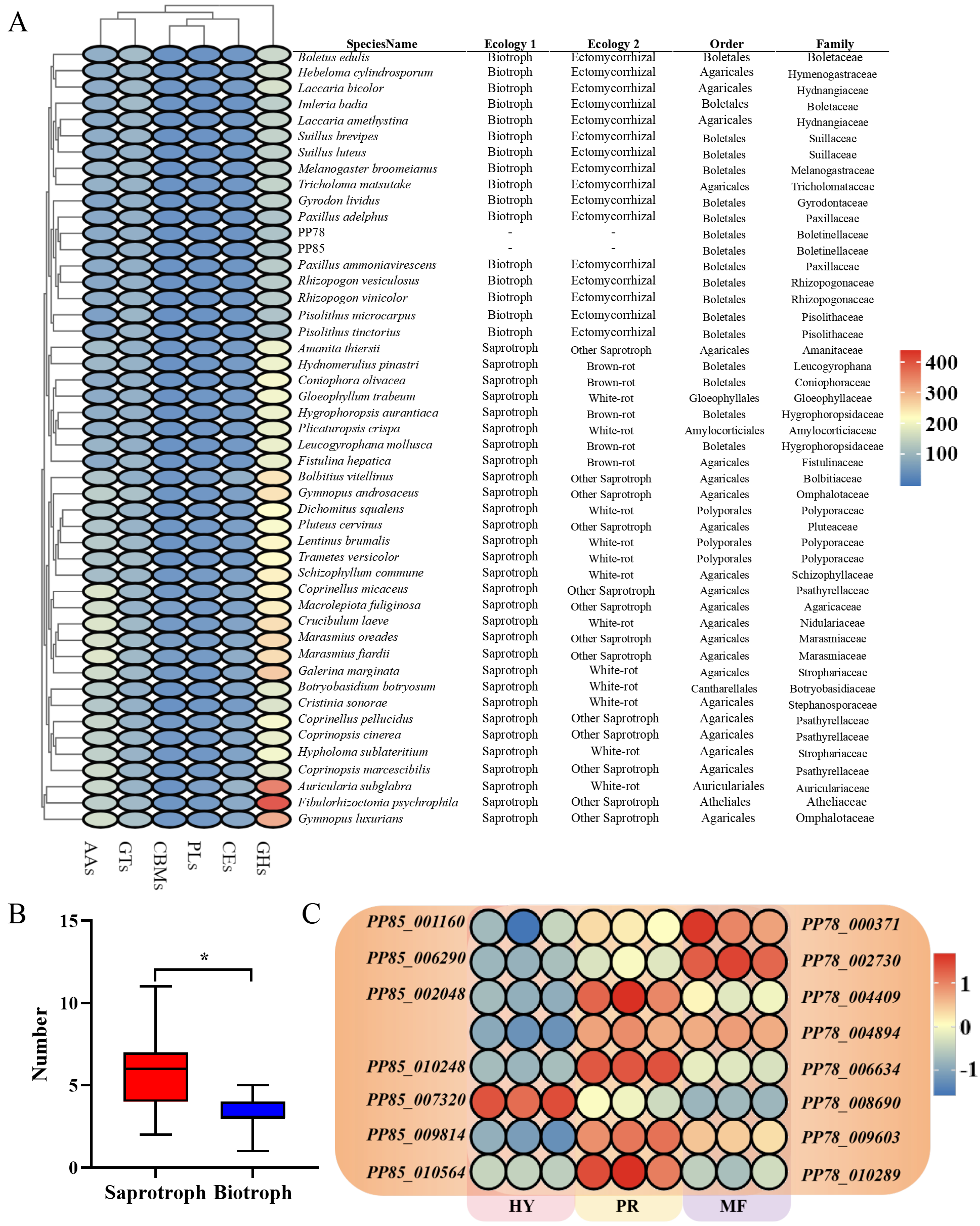


**Figure S19.** Carbohydrate-degrading enzyme analyses in *Phlebopus portentosus* and other Agaricomycotina fungi **A** Heatmap of carbohydrate-degrading enzyme families in *Phlebopus portentosus* and other Agaricomycotina fungi; **B** GH31 family gene copys analysis in Saprotroph and Biotroph in Agaricomycotina fungi; **C** Transcriptional profiles of GH31 genes across different developmental stages in *Phlebopus portentosus.* HY: hypha; PR: primordium; FB: fruiting bodies
